# Brain haemorrhage detection using a SVM classifier with electrical impedance tomography measurement frames

**DOI:** 10.1371/journal.pone.0200469

**Published:** 2018-07-12

**Authors:** Barry McDermott, Martin O’Halloran, Emily Porter, Adam Santorelli

**Affiliations:** Translational Medical Device Lab, National University of Ireland Galway, Galway, Ireland; University of Craiova, ROMANIA

## Abstract

Brain haemorrhages often require urgent treatment with a consequent need for quick and accurate diagnosis. Therefore, in this study, we investigate Support Vector Machine (SVM) classifiers for detecting brain haemorrhages using Electrical Impedance Tomography (EIT) measurement frames. A 2-layer model of the head, along with a series of haemorrhages, is designed as both numerical models and physical phantoms. EIT measurement frames, taken from an electrode array placed on the head surface, are used to train and test linear SVM classifiers. Various scenarios are implemented on both platforms to examine the impact of variables such as noise level, lesion location, lesion size, variation in electrode positioning, and variation in anatomy, on the classifier performance. The classifier performed well in numerical models (sensitivity and specificity of 90%+) with signal-to-noise ratios of 60 dB+, was independent of lesion location, and could detect lesions reliably down to the tested minimum volume of 5 ml. Slight variations in electrode layout did not affect performance. Performance was affected by variations in anatomy however, emphasising the need for large training sets covering different anatomies. The phantom models proved more challenging, with maximal sensitivity and specificity of 75% when used with the linear SVM. Finally, the performance of two more complex classifiers is briefly examined and compared to the linear SVM classifier. These results demonstrate that a radial basis function (RBF) SVM classifier and a neural network classifier can improve detection accuracy. Classifiers applied to EIT measurement frames is a novel approach for lesion detection and may offer an effective diagnostic tool clinically. A challenge is to translate the strong results from numerical models into real world phantoms and ultimately human patients, as well as the selection and development of optimal classifiers for this application.

## 1. Introduction

Clinically important conditions such as stroke and traumatic brain injury may feature brain haemorrhage as part of the patient presentation. The ability to definitively rule in or rule out the presence of a haemorrhage in these conditions is vital to the subsequent patient path, and therefore to the resulting patient outcomes.

Stroke is responsible for about 9% of global deaths and is the 2^nd^ most prolific killer after heart disease [1]. In survivors, there is significant incidence of morbidity with irreversible loss of neurological tissue resulting in conditions such as paralysis and dysphagia. Approximately 500 out of 100,000 people live with the consequences of stroke and it consumes 2–4% of global health budgets [1]. The cause of stroke is either a haemorrhagic or an ischaemic lesion. Treatment is radically different depending on the stroke type, necessitating absolute differentiation between the types [1,2]. A key treatment for ischaemic stroke, alteplase, is only licensed for use within 3 hours of ictus in the US (and within 4.5 hours in the UK), and due to the pharmacological nature of the drug, can be fatal if given to a haemorrhagic stroke patient [1,3]. These constraints underline the need for rapid diagnosis. Neuroimaging by Computed Tomography (CT) and Magnetic Resonance Imaging (MRI) are the gold standard imaging modalities used, and are excellent at imaging acute haemorrhage, while ischaemia often takes longer to be detectable [4]. As such, detecting the presence or absence of haemorrhage may be sufficient to initiate appropriate treatment. Detection of a bleed categorises the patient as having a haemorrhagic stroke, whereas the absence of a bleed de-facto categorises them as having an ischaemic stroke [5].

Traumatic Brain Injury (TBI) can result from any external force impacting on the head. 1.6 million incidents of TBI occur annually in the US resulting in 50,000 deaths and 70,000 patients left with permanent neurological defects [6]. Significantly, TBI accounts for 10% of the annual US healthcare budget [7]. TBI covers a range of injuries from minor to major. The latter category includes haemorrhage. Currently, physical examination using standardised tests like the Glasgow Coma Scale (GCS) are used to decide on whether to include CT imaging in a patient workup, the latter indicated for conditions such as haemorrhage. Again, the ability to rule in or out haemorrhage in TBI patients early into an assessment would allow more efficient use of CT resources and better patient outcomes [6]. The two clinical examples described above highlight the need for a sensitive and specific technique for the detection of brain haemorrhage, that would satisfy the constraints for usage in a timely manner (and hence ideally portable for paramedic use) and cost-effectiveness.

### 1.1 Electrical impedance tomography

Electrical impedance tomography (EIT) is an imaging modality that may facilitate the development of a sensitive, specific, rapid, portable and cost-effective brain haemorrhage detection medical device. EIT systems are low-cost and compact. It has the further advantages of being non-invasive and hazard free [8]. EIT has been used most successfully in monitoring time-changing scenes such as lung function applications. However, to date, EIT systems have struggled in imaging static or quasi-static scenes [9], which would be the case with brain haemorrhage. EIT involves the placement of electrodes around the region of interest, with alternating current injected between electrode pairs of a frequency typically on the order of 1 kHz– 1 MHz, and voltages measured between other pairs in a precise pattern. A set of measurements from all injection-measurement electrode combinations constitutes a complete measurement frame. Production of an image of the volume encompassed by the electrodes typically follows, this image being a map of the electrical conductivity of the tissues contained in the volume. Image reconstruction algorithms often operate based on differences between sets of measurement frames and thus can struggle with static scenes [8–10].

### 1.2 Machine learning applied to EIT measurement frames

This study is designed as an initial proof of concept to examine if EIT measurement frames can directly be used in conjunction with machine learning algorithms to detect brain haemorrhages, without the complexity of reconstructing an image. Persson et al. have had success using microwave scattering measurements for a similar purpose [11]. To our knowledge EIT measurements have not been used in this application to any extent, although our group has conducted some early research into the problem [12,13]. Electrical conductivity or impedance, the physical parameter indirectly measured with EIT, does vary detectably between normal brain parenchyma and haemorrhage [14,15], and thus the corresponding EIT measurements frames should have detectable differences.

Support Vector Machine (SVM) is a machine learning algorithm that is typically implemented for binary classification. SVMs operate on the principle of finding a hyperplane that optimally separates data points from differing classes. The nature of the hyperplane is defined by the kernel employed by the SVM, with a linear kernel being one of the simpler examples [16]. Indeed SVMs have been used in similar studies to this, examples including microwave measurements applied to breast cancer detection [17], [18], and other electrical impedance paradigms applied to various tissues [19–22]. In [19], electrical impedance measurements from a multi-frequency sweep (electrical impedance spectroscopy, EIS) of different tissues were used to train and test a support vector machine (SVM) to classify ‘breast’ and ‘not breast’. Sensitivity and specificity was reported at ~80%. EIS was again used in [20] to measure impedance properties of breast tissue to identify patients at risk of cancer using SVMs. In this study, an area under the receiver operating characteristic (ROC) curve of 0.816 was reported. A similar technique and application was reported in [21]. In, [22] SVM models were used with impedance measurements to improve the sensitivity of prostate biopsies to abnormal tissues in the region of the biopsy needle. Finally in [23], EIT measurement frames were used to estimate bladder volumes through machine learning which estimated the volume without the need for an image.

In this study, we investigate the use of a linear SVM model on EIT data from the head. EIT measurement frames from a range of different numerical models, along with a physical phantom, are used. These models are designed as a simplified anatomically precise 2-layer head with an inner brain layer and an outer layer of aggregate tissues external to the brain. These models were tested with and without a variety of haemorrhagic lesions under a range of experimental conditions designed to examine and quantify the performance of the classifier when discrete variables are isolated and changed, including those of noise level, lesion location, lesion size, changes in electrode positioning, and variation in head anatomy. As a final study, the classifier is trained and tested with variants in all of the possible parameters. Due to the flexibility in creating numerical models, more tests are performed with this platform, but a significant amount of the work was replicated on the physical phantom platform as well.

The subsequent layout of the paper is as follows. Section 2 introduces SVMs and describes performance metrics which are applied to results and reported throughout this study, Section 3 outlines the numerical models, EIT setup used, and simulations, with the results presented and discussed. Then, Section 4 introduces the physical phantom, describes the experimental measurements, and presents the phantom-based results. Section 5 examines the performance of two different classifiers, in addition to the linear SVM, on the full numerically derived data set varying all of the parameters, and on the corresponding experimental data set. Although this work is an initial proof of concept for applying machine learning techniques to EIT measurement frames, it is also useful to examine the performance of other classifiers to support the ultimate choice of classifier for this application. Finally, the paper concludes with Section 6, which provides a discussion on the relevance of the findings, limitations of the study, and future work. This section also highlights the significance of this approach as a promising way to couple EIT to classification for important clinical applications.

## 2. Support Vector Machines

**Fig 1:**
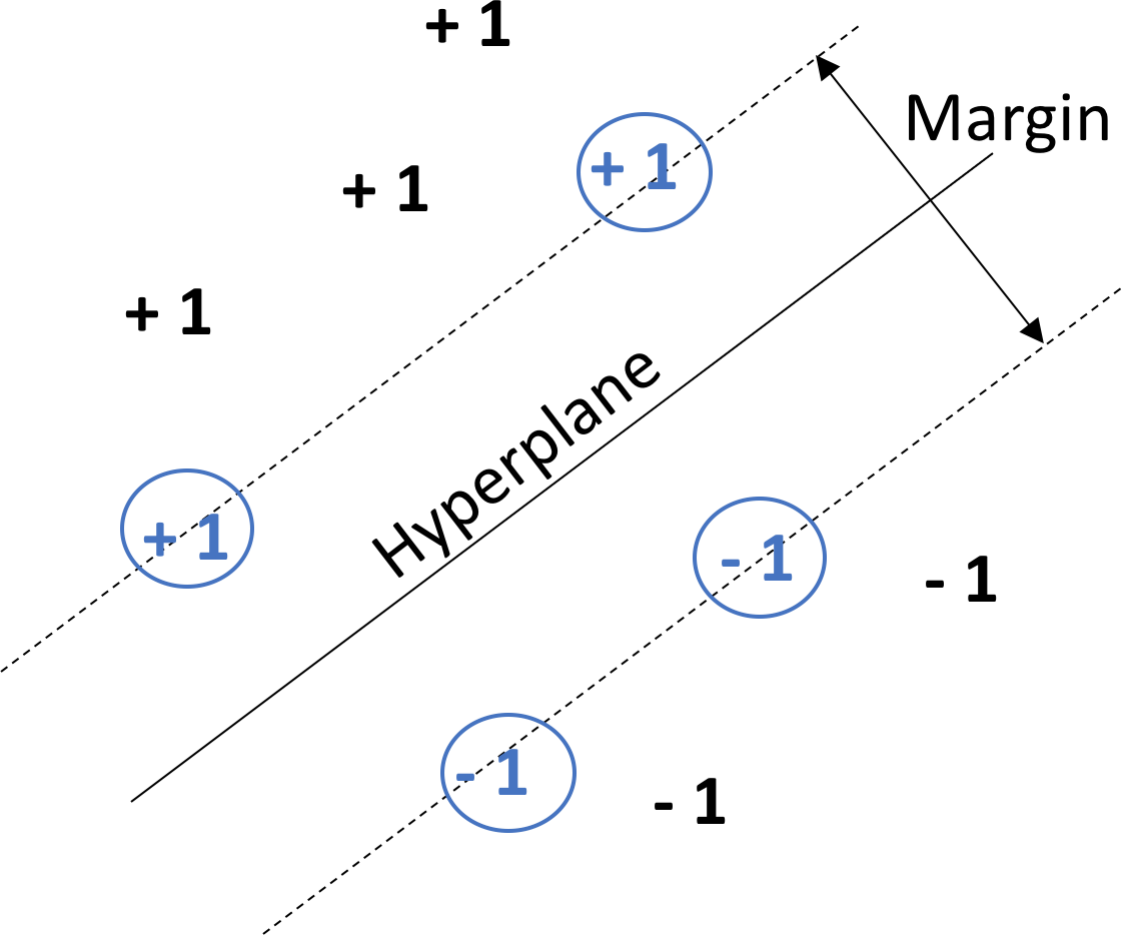
The basis of SVM classification. Here *n*-dimensional data points are separated by a 2-dimensional hyperplane into +1 or –1 categories depending on what side of the hyperplane they are on. Support vectors are those data points that define the margin about the hyperplane, these support vectors are shown in blue in circles.

The extensive use of Support Vector Machines in previous studies involving biomedical signals [17–23] motivated the choice of using this type of classifier. SVMs were originally designed for binary classification. Binary classification is employed here, a case with a haemorrhage being labelled as +1 and a case without labelled as –1. SVMs classify by calculating the hyperplane that optimally separates the training data points belonging to the two classes. Data points are assigned into one of the two categories depending on which side of the plane they lie on. This hyperplane will incorporate the widest margin possible with the data points that form the border of the margin on each of the +1 and –1 sides called the support vectors [16]. An example of this hyperplane and separation of classes is shown in Fig 1, with observations consisting of *n*-dimensional data points separated by a 2-dimensional hyperplane (a line). In the case of an EIT measurement frame from a 16-electrode ring, each measurement frame (observation) will be a 208-dimensional data point or vector [8].

The data points for a given experiment are divided into training and test sets. The training set will contain data points labelled with the correct classification of ±1. This training set is used to create a SVM model, which, as part of modelling, finds and defines the hyperplane in multidimensional space that best separates the training data. During the modelling process, ten-fold cross validation and hyper-parameter optimisation, using a Bayesian optimisation protocol, is performed to ensure an optimally trained classifier. In this study, a linear SVM is used, meaning that the hyperplane is linear in nature. After the model is created, unseen testing data is classified by the SVM. The classification results of the trained SVM model on this unseen test data are used to describe performance. These performance results are reported here primarily as the sensitivity and specificity of the classifier on this test data. Another parameter of interest is the receiver operating characteristic (ROC) curve of the model, reported indirectly as the area under the ROC curve (AUC). The AUC values reported here are those for the ROC curve of the trained model, that is the ROC curve produced from comparing the true labels of the training data to the predicted labels assigned to the training data by the model. Throughout the paper it is the performance on the unseen test data of the trained classifier that is reported as the results for a given study. The software package MATLAB and the MATLAB statistics and machine learning toolbox is used throughout for the training and testing of the linear SVMs [24].

In our clinical application, the key objective is to definitively detect haemorrhage. Ideally, the classifier would be 100% sensitive and 100% specific to haemorrhage, as would be the case where the AUC = 1. However, in cases where this is not possible, that is, where the ROC curve indicates an imperfect classifier, then the priority is to maximise sensitivity at the expense of decreased specificity. In the case of stroke, for example, reduced specificity means an increased rate of false positives (i.e., patients who have normal healthy brain being diagnosed as having a bleed). Although not ideal, the converse, reduced sensitivity, would mean increased false negatives, leading to haemorrhagic patients being misdiagnosed as being bleed-free and potentially receiving alteplase, which may be fatal. In traumatic brain injury, increased sensitivity means haemorrhagic patients are not missed and are properly referred for CT scanning with the consequent reduction in specificity resulting in some non-haemorrhagic patients also receiving CT scans. This result is preferable to the alternative of some haemorrhagic TBI patients having false negatives and not receiving an appropriate scan. Therefore, the ROC curve (if the AUC is not 1) can be used to choose an ideal operating point of the SVM classifier, resulting in changes to the sensitivity and specificity balance. As part of the classification process, the trained SVM model calculates the posterior probability of the test case belonging to both classes. The default threshold, called the ‘default operating point’ here, is 0.5. If the probability of the case belonging to the –1 category is greater than or equal to 0.5, it is classified as –1, otherwise it is classified as +1. We classify, and report performance results at both this default operating point and at an adjusted operating point designed to maximise sensitivity. The adjusted operating point is selected from finding the first point on the ROC curve which has a true positive rate of 1.0. Use of the adjusted operating point effectively increases the threshold to a value greater than 0.5, thus boosting sensitivity to bleeds at a cost to specificity.

Finally, as described above, for comparison purposes, the performance of two different classifiers, a SVM using a Radial Basis Function (RBF) kernel and a Neural Network (NN), was also examined with both numerical and phantom data. The comparison between these three classifiers is discussed in Section 5.

## 3. Classification experiments on numerical models

This section begins with a description of the numerical models created for this study, along with a brief description of the EIT setup used. Following this, each of the series of experiments performed using this test platform is outlined with results presented and discussed. In total, 6 sets of experiments were performed, the first 5 isolating and studying the individual effect of a key variable on the classification results. These 5 variables included noise contaminating the measurement frames, differences in lesion location, differences in lesion size, changes in the placement of the electrode array, and differences in head anatomy. Within a given experiment, all other variables are held constant unless noted. Lastly, a 6^th^ and final experiment trained and tested the classifier on cases varying all 5 of these variables.

### 3.1 Numerical models

The model used in the study was an anatomically accurate but simplified model of the human head comprised of 2-layers: an inner brain layer and an outer aggregate layer of tissues external to the brain. Anatomically accurate stereolithography (STL) files of the human head and brain were used to create this 2-layer model as a finite element model (FEM). The head STL file was reverse engineered from a polygon mesh [25] while the brain STL file was developed from MRI studies [26]. These two STL files were called the “base” STL files. The rationale for the 2-layer simplification was the exploratory nature of this work and the fact that this simplification allowed fabrication of a physical phantom nearly identical to the base numerical model.

The EIT setup chosen was that of a 16-member electrode ring placed symmetrically across the sagittal plane at a variety of heights, all forming a plane from about the mid forehead sweeping around to the inion at the rear of the head. The precise pattern of EIT injection and measurement pairs resulting in a frame was a so called “skip 2” pattern, with measurements not taken from the injecting electrodes, as is conventional [8,9]. The resultant frames are inputted into the classifier unaltered or sorted by voltage measurement value. Sorting was undertaken as it was hypothesized that it may improve performance in some scenarios, particularly in those test cases featuring lesions in locations not seen in the training data. The rationale for this is described later in the next two sections.

In order to numerically model different head and brain anatomies, these base STL files were both increased and reduced in size by 5% in each of the X, Y, Z axes, respectively, as well in all 3 axes simultaneously, using the Autodesk Fusion 360 computer aided design (CAD) package [27]. The ±5% variance was based on work studying adult head circumferences [28] and assumed that variances in circumference will extrapolate to head size and brain size. These variations resulted in 9 different models for each the head and the brain, with 81 different head and brain combinations possible. It is recognised that combinations of, for example, +5% head size coupled with –5% brain size may be unlikely in reality, but such extremes were used here for completeness.

The STL files were meshed into a FEM using EIDORS [29] aided by Netgen [30] and Gmsh [31]. During meshing, the 16-member electrode ring is also positioned on the exterior of the model, with the mesh refined in the areas where the electrodes contact [32]. The electrode ring was placed at 3 possible heights, differing by 2 mm from each other, to mimic user error in placing electrodes in repeated experiments. Electrode modelling errors such as these are a key challenge in EIT [33]. All possible combinations of head, brain and electrode layout were meshed resulting in 243 normal numerical head models. The head model produced from the base STL files of the head and brain combined with the electrode ring at the middle of the 3 heights was called the “base numerical model” as it was used as the standard in many of the experiments.

The haemorrhages were modelled as simple spheres, generated using the Fusion 360 software. These spheres varied in volume. A 30 ml bleed is often seen as a threshold indicator of worse outcomes in stroke patients [34], thus bleeds of this size or larger (60 ml) were chosen for the initial experiment; a second experiment consisted of classifying smaller (5 ml, 10 ml, and 20 ml), more difficult to detect bleeds. The spheres were placed in different locations: the 4 cardinal points of north (front), south (back), east (right side) and west (left side) in the plane of the electrode rings and inside but towards the exterior surface of, and with respect to, the FEM of the brain.

Each of the 60 ml and 30 ml bleeds, at each of the 4 locations were meshed with each of the 243 possible normal models to form 1,944 separate lesion-affected head models, with each given model having 1 lesion.

The electrical conductivity of a tissue is the fundamental property that affects EIT measurements, and which is traditionally captured in EIT image reconstruction. Hence, realistic conductivity values are assigned to the layers of the model with the outer layer given a value of 0.1 S/m, the brain layer set at 0.3 S/m, and the haemorrhagic lesions 0.7 S/m, based on the work done in [35]. In a given model, EIDORS was used to specify the “skip 2” stimulation and measurement pattern and used to calculate a measurement frame for the setup. The recorded voltages at these electrodes, and each resultant measurement frame, were then used to train and test the linear SVM classifiers used.

**Fig 2:**
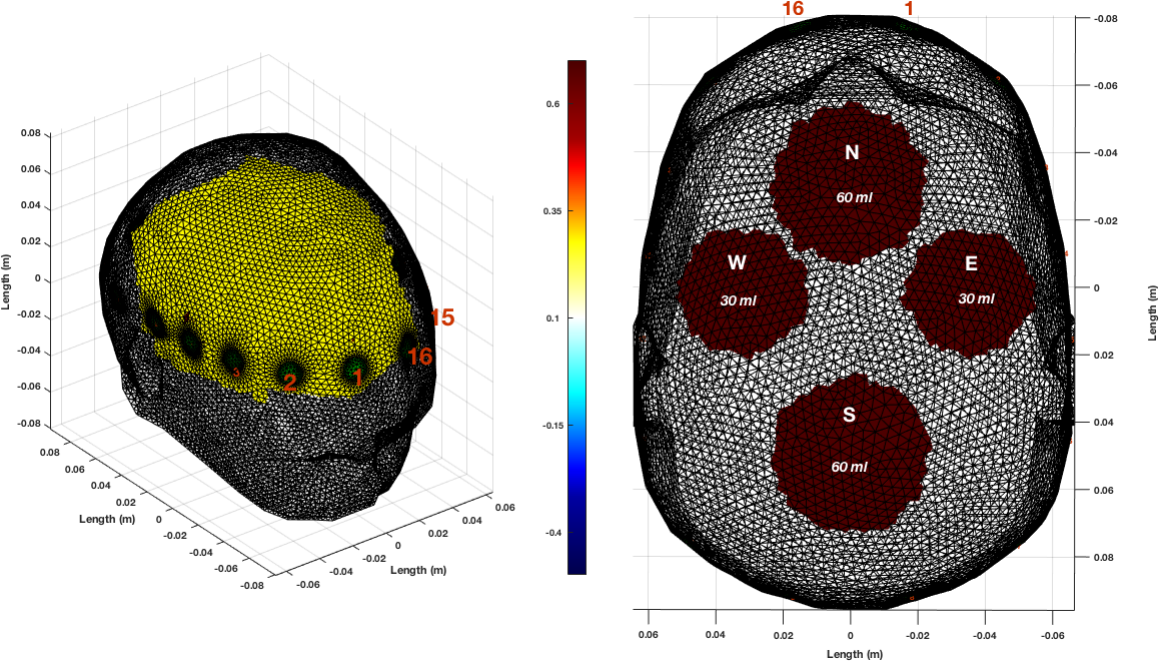
Left: The base numerical model comprised of the normal sized head and brain is shown as a FEM with a 16-member electrode ring. The electrode contact areas are shown in green with some of the electrodes numbered (red) for clarity. Right: Bleeds may be positioned at one of 4 possible locations, north (front), south (back), east (right) or west (left) in the plane of the electrode ring and inside but approaching the exterior surface of the brain layer. The brain layer is hidden in this image facilitate visualisation of the lesions. The two bleed volumes shown are 30 ml and 60 ml. Two of the electrodes are again numbered for orientation. Also shown is a scale of assigned conductivity values, with the outer layer shown as white/transparent (0.1 S/m), the brain layer as yellow (0.3 S/m), and the haemorrhagic lesions as burgundy (0.7 S/m).

In Fig 2, the base numerical model with the electrode ring is shown, as well a model with the brain layer hidden and 4 bleeds illustrating the 60 ml and 30 ml sizes and the 4 possible locations.

### 3.2 Study 1: Effect of noise

The performance of each SVM classifier is assessed at different signal-to-noise ratios (SNR). The base numerical model is used as the normal with two lesion models: the normal with either the 30 ml or 60 ml bleed in the north location. Measurement frames are generated for each of the 3 models and noise is added using EIDORS to achieve each specified SNR. A linear SVM was trained with 500 frames (250 normal and 125 from each of the two lesion models) and tested with 200 unseen frames (100 normal and 50 from the two lesion models). The training and testing was performed at each of 80 dB, 60 dB, 40 dB and 20 dB noise levels. Some EIT applications such as thoracic imaging can operate sufficiently well with an SNR of 30–40 dB whereas others, particularly those trying to detects neural signals and facing related issues such as the dampening effect of the skull require systems with an SNR of 80 dB or higher [36]. The SNR values used here were selected to cover this range and to see the effect of data noisier than that usually used.

**Fig 3:**
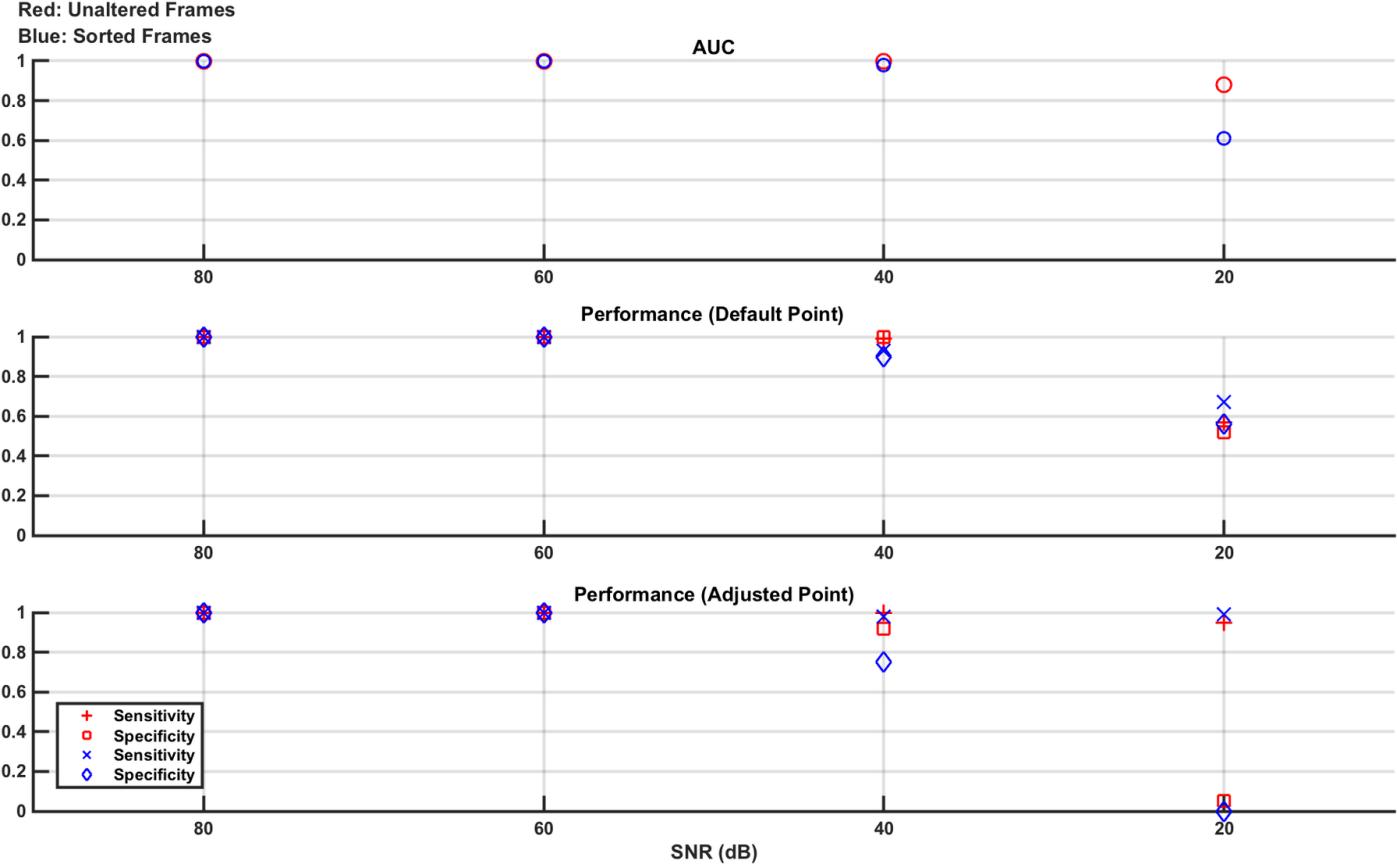
Classifier results from test data in numerical model Study 1: Effect of noise. Separate experiments are done for 80 dB, 60 dB, 40 dB, and 20 dB noise levels. The measurement frames used to train and test the linear SVM are unaltered (red) or sorted by value (blue). For both of these resultant classifier models, the performance is given as the AUC of the ROC curve generated from the training data and the sensitivity and specificity of the classifier on the test data at the default operating point and at the adjusted operating point (selected to augment sensitivity, at the expense of specificity). From the plots, it is seen that the classifiers perform well at 80 dB and 60 dB with performance starting to drop at 40 dB and becoming poor at 20 dB. The adjusted operating point does boost sensitivity as expected, with the most obvious benefit seen at 40 dB.

As detailed above, the measurement frames are either unaltered or sorted by value (low voltage to high voltage). Hence, each SNR level has two corresponding SVM models trained and tested using these two frame types, each with an AUC reported. In addition, the sensitivity and specificity for each classifier at each SNR level has two performance values: that when tested at the default operating point and then when retested at the adjusted operating point designed to maximise sensitivity. These results are shown in Fig 3.

The results of Fig 3 show that the SVM classifier trained, and tested, with both the unaltered and sorted measurement frames performs well, with an AUC, as well as sensitivity and specificity, at or near 1, for the 80 dB and 60 dB noise levels. At 40 dB, the classifier performance significantly degrades and the benefit of the adjusted operating point compared to the default operating point is seen, as sensitivity is boosted at the expense of specificity. For example, in the sorted frame results, the sensitivity jumps from 0.92 to 1.0 while the specificity drops from 0.9 to 0.78. The performance at 20 dB is poor and there is no difference in the performance between using the unaltered or the sorted frames. The SNR value of 20 dB was chosen as an extreme value, significantly lower than that used even in high contrast time changing scenes such as thoracic EIT [36]. As such, the poor performance reported at this value is expected and helps put a lower limit on the acceptable SNR range that can be used.

**Fig 4:**
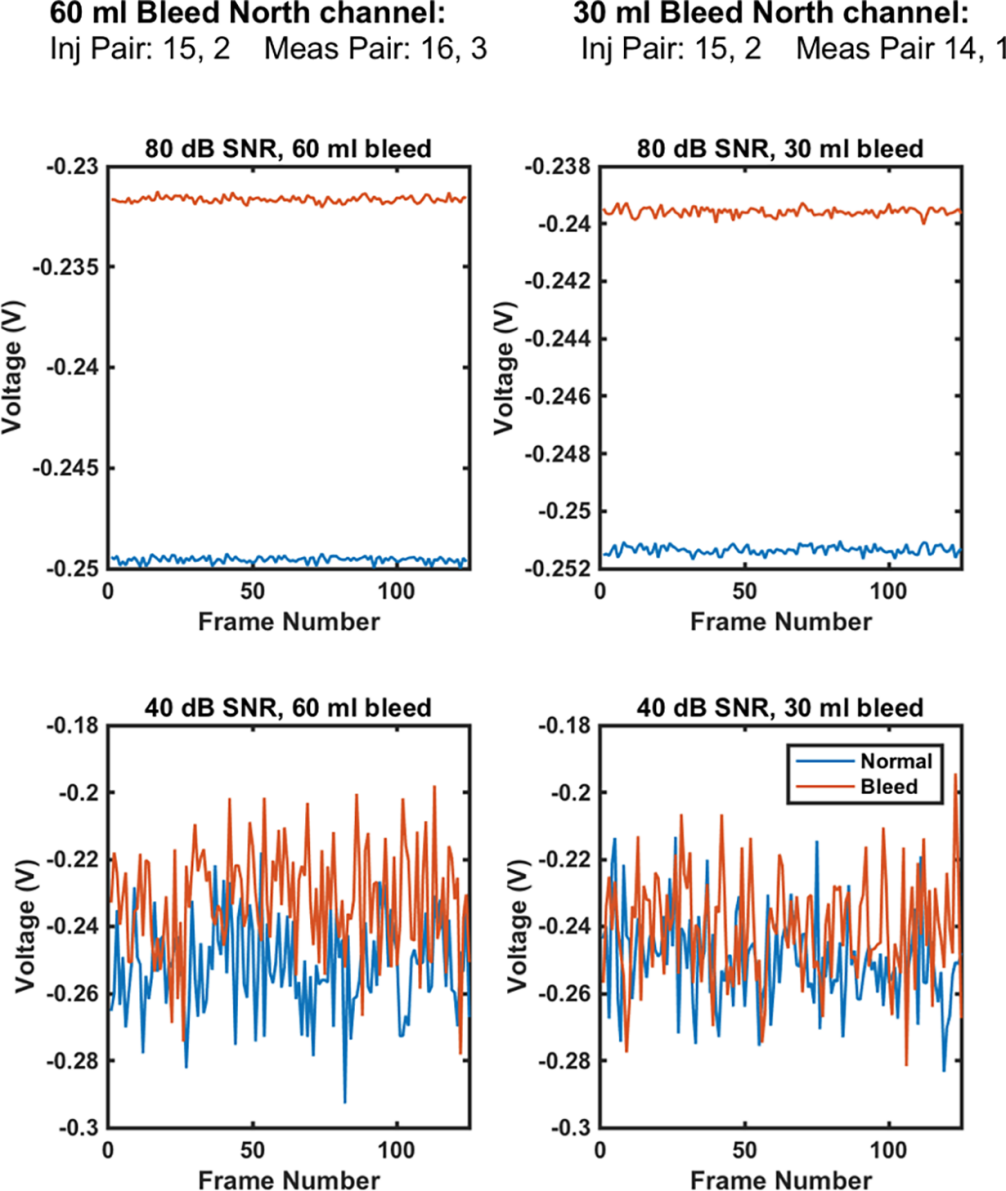
The channels of maximal difference between the normal and lesion cases are reported. The channels imply the lesion is at the north location, as electrodes #1 & #16 are adjacent to this location. The 60 ml bleed shows a greater difference relative to the normal than does the 30 ml case. Further, separation is more distinct at a higher SNR, with overlap increasing at 40 dB.

It was of interest in this part of the study to analyse if the measurement frames could be used to detect lesion location and size. A haemorrhagic lesion has a higher conductivity than the surrounding brain. Hence, a given channel of the measurement frame, i.e., one specific combination of injecting and measurement electrode pairs, would be expected to give a lower measured voltage magnitude for the fixed injection current if the lesion is near to the electrodes involved in the channel. The channel that had the maximal average difference in value between the normal frames and the lesion frames was calculated for both cases of the 30 ml and 60 ml haemorrhage in the north location. The electrode layout used meant that electrodes 1 & 16 lie in front of the north location with 2 & 15 also nearby and so on. These maximal difference channels are shown in Fig 4. These maximal difference channels, as expected, feature electrodes directly adjacent to the lesion location. It would also be expected that the magnitude of the difference would be greater for the 60 ml bleed than the 30 ml bleed compared to the normal and these differences should be clearer at higher SNR. Fig 4 confirms these hypotheses with plots of voltage measurements at the maximal difference channel shown for the 80 dB and 40 dB cases. The separation is indeed greater for the 60 ml bleed and the separation is more distinct for the 80 dB SNR case.

### 3.3 Study 2: Effect of lesion location

**Fig 5:**
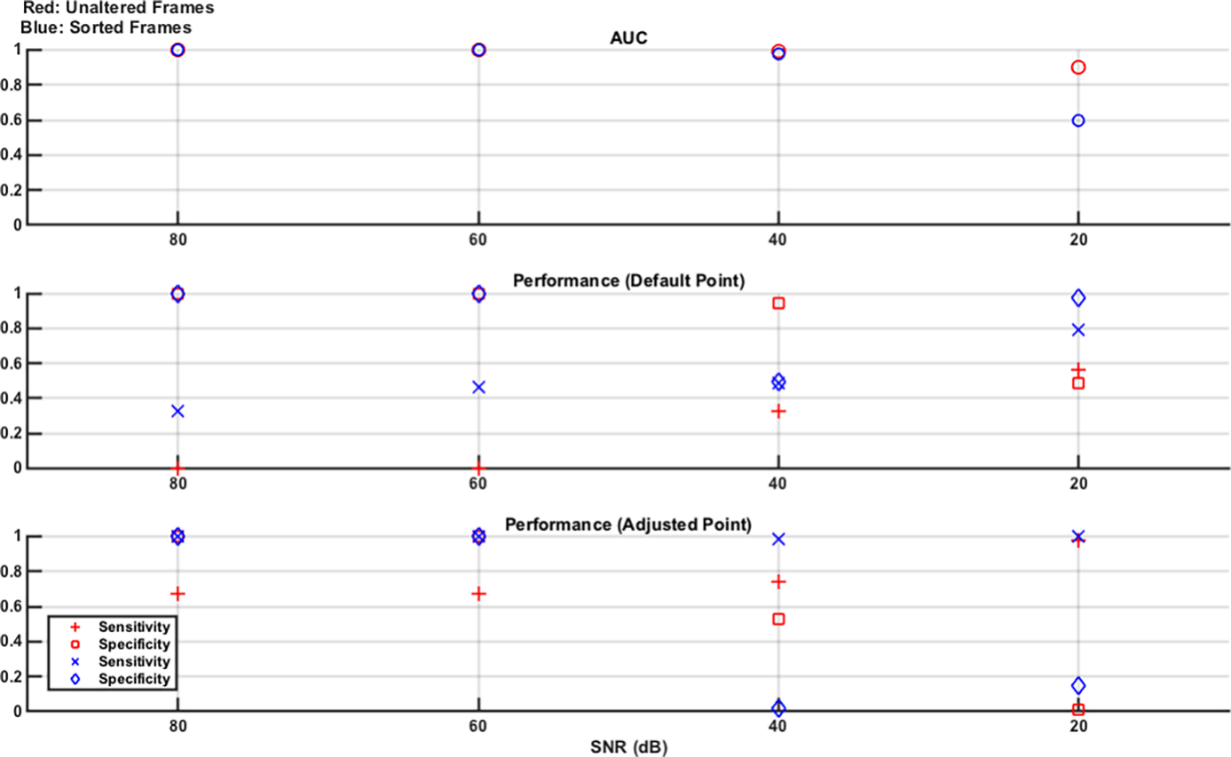
Classifier results from test data in numerical model Study 2: Effect of lesion location. Separate experiments are done for 80 dB, 60 dB, 40 dB and 20 dB noise levels with results arranged in the same format as Fig 3. Both using a sorted measurement frame and the adjusted operating point dramatically improve sensitivity at a given SNR. Implementing both of these modifications result in excellent classifier performance at the 80 dB and 60 dB points, indicating that in these cases the classifier still works well even when test data lesion locations differ from training data locations.

The classifier performance is assessed with test data that includes lesions at positions different to those used in the training data. The base numerical model is again used as the normal, with both the 30 ml and 60 ml lesions added to the north location separately. These models are used to train the classifier with 500 measurement frames (250 from the normal and 125 each from the two lesion models). The performance is then tested with 120 unseen normal measurement frames and 20 measurement frames each from 6 lesion models created from all individual combinations of the normal with east, south, west and 30 ml or 60 ml. Hence, the classifier is tested on lesions in locations that were not included in the training set. The results are presented as before–two classifiers are trained depending on whether the measurement frames are unaltered or sorted, giving two separate AUC values from the respective ROC curves generated from the training data, then the sensitivity and specificity for each classifier on the test data is reported at both the default operating point and at the adjusted operating point (selected for better sensitivity). The experiment is repeated for each of 80 dB, 60 dB, 40 dB and 20 dB simulated noise levels, with the results shown in Fig 5.

If the measurement frames are left unaltered and classification is performed at the default operating point, then sensitivity is low at all SNRs: at about 0 for 80 dB and 60 dB and paradoxically highest at about 0.55 for 20 dB. Moving to the adjusted operating point (still with unaltered measurement frames) helps to improve sensitivity, with values now above 0.6 in all cases, but it is still unacceptably low. In this case, the classifier cannot detect lesions when the location of the test case is one the classifier is not trained on. Specificity is not as affected as this is primarily a measure of ability to detect the ‘-1’ class of normal which the classifier is trained and tested on.

However, the simple pre-processing step of sorting the measurement frame values from low to high helps boost sensitivity at all SNR levels compared to the respective unaltered case. The purpose of sorting the frames is to help the classifier in correctly classifying lesions at locations not seen previously in the test set. As described in the previous section, the presence of the lesion should result in lower magnitude voltage measurements especially for those channels in the locality of it. In the normal case, sorting should not overly affect the frame, at least in this simple model where electrode distance and regional differences in tissues are neglected. In the case of a lesion, sorting should result in a similar resultant frame regardless of lesion location. The results shown in Fig 5 provide evidence supporting this hypothesis. The adjusted operating point further boosts sensitivity, with excellent performance of sensitivity and specificity both equal to 1, seen at both 80 dB and 60 dB when the measurement frames are sorted. If frames are sorted and the adjusted operating point is chosen, then the classifier seems to be independent of the lesion locations used, for the 60 dB and 80 dB SNR levels.

### 3.4 Study 3: Effect of lesion size

**Fig 6:**
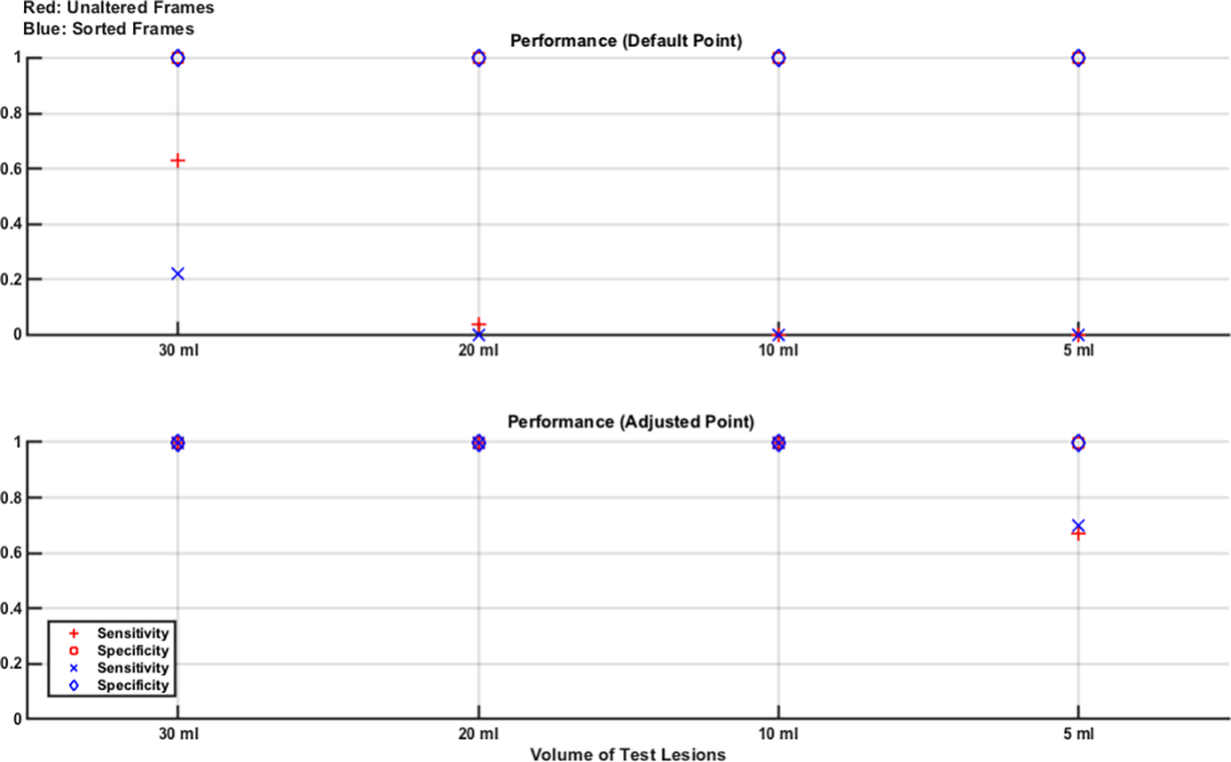
Classifier results from test data in numerical model Study 3: Effect of lesion size. The models are trained using the 60 ml lesion in all possible locations in the base numerical model. Separate testing is then performed on unseen normal frames and separately each of 30 ml, 20 ml, 10 ml and 5 ml lesion at all locations, with 60 dB noise added in all cases. Training data features all locations, rendering the effect of sorting the frame redundant. Using the adjusted operating point boosts sensitivity in all cases with lesions robustly detectable down to a 10 ml volume.

Next, the classifier is tested on data sets with lesions of sizes not used in the training set, with a fixed SNR of 60 dB. The base numerical normal model is used to produce 300 noisy measurement frames. Next, 4 lesion models are produced using this normal model along with the 60 ml lesion at each of the 4 locations. 75 measurement frames from each lesion model are produced. This combined 600 measurement frame set is used as before, with an SVM classifier trained with both unaltered and sorted frames. The resultant classifiers both had an AUC value of 1. Next, the models are tested using 80 unseen normal frames and 80 unseen frames from the 30 ml lesion positioned at each of the 4 locations (20 frames from each location). The testing is repeated in an identical way for each of 20 ml, 10 ml and 5 ml model lesions. Hence, the models are trained on the largest lesion and tested sequentially on each of the smaller lesions. The classifier performance results for sensitivity and specificity are given in Fig 6.

**Fig 7:**
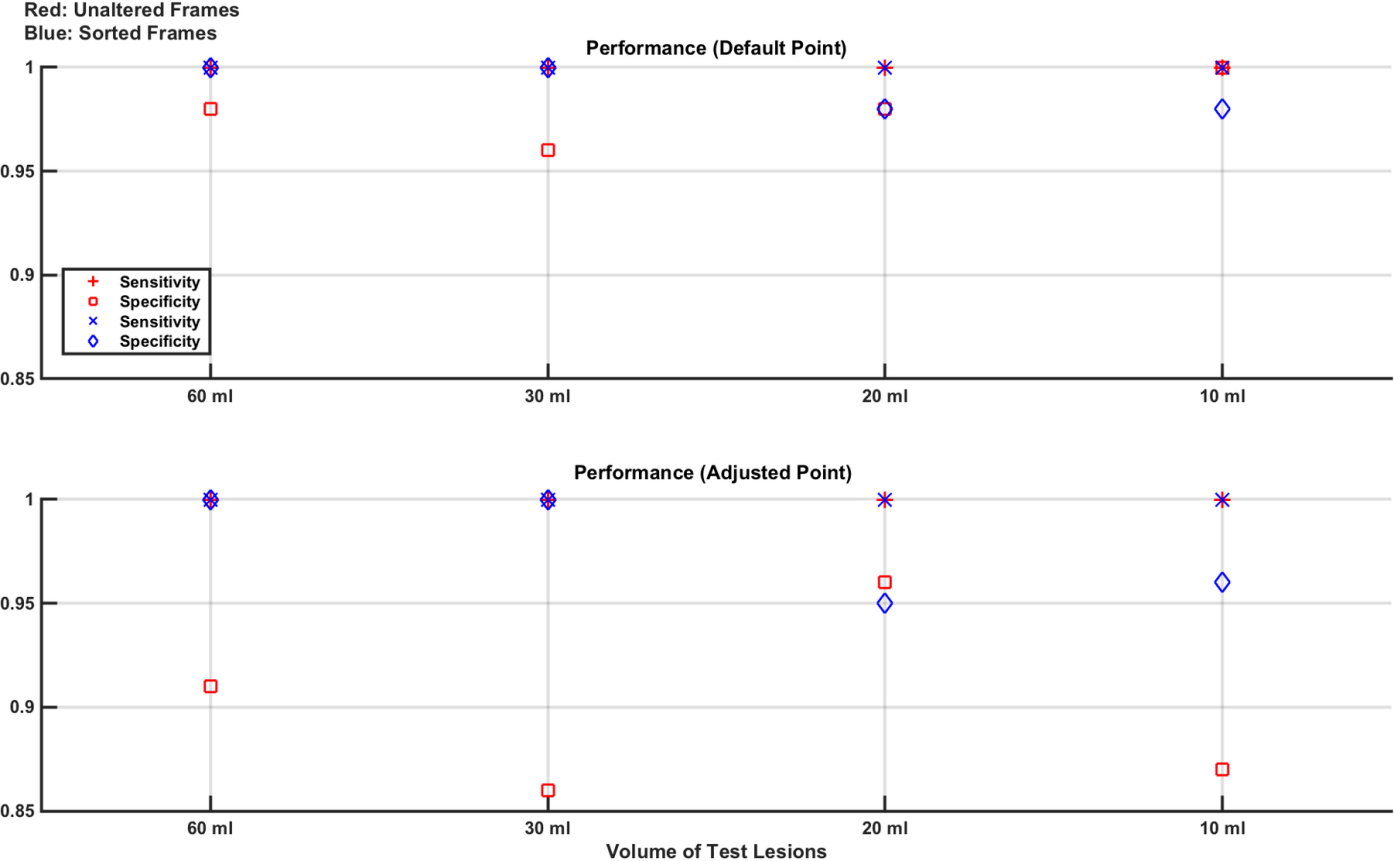
Classifier results from test data in numerical model Study 3: Effect of lesion size. The models are trained using the 5 ml lesion in all possible locations in the base numerical model. Separate testing is then performed on unseen normal frames and separately each of 60 ml, 30 ml, 20 ml and 10 ml lesion at all locations, with 60 dB noise added in all cases. Training data features all locations, rendering the effect of sorting the frame redundant. Sensitivity and specificity in all cases is excellent at the default operating point, while using the adjusted operating point unnecessarily reduces specificity of the classifier that uses unaltered frames but does not appreciably affect performance of the classifier that uses sorted frames.

Finally, the entire experiment is repeated, this time training on the smallest lesion (5 ml) and testing on the 60 ml, 30 ml, 20 ml and 10 ml cases (as well as the normal being used throughout). The trained classifiers both reported an AUC of 1 again. These testing results are given in Fig 7.

The results in Fig 6 indicate that when the classifiers are trained using the 60 ml lesion there is an inability to detect smaller test lesions unless the adjusted operating point is used. Since training featured cases from all locations, the benefit of the sorted frame, which was very effective in the previous study on effect of location, is redundant here. If the adjusted operating point is used, increasing sensitivity, then robust classification (sensitivity and specificity both of 1) down to a 10 ml volume is achieved. In Fig 7, the 5 ml lesion is used in training and there is improved performance over Fig 6, with all larger test lesion sizes being detected at the default operating point. Changing to the adjusted operating point unnecessarily decreases specificity with unaltered frames, although sorted frames are more robust to this effect giving nearly the same performance at both operating points. In this case, sensitivity of 1 was achieved at the default operating point using the default threshold. Moving to the adjusted point, which forces the threshold up, unnecessarily increased the number of false positives with no reduction in false negatives possible. In cases like here where the default threshold of 0.5 already gives a sensitivity of 1, the adjusted threshold is also 0.5. However, the adjusted threshold now categorises cases with an equal probability of being +1 or -1 as being +1 (bleed) whereas the default threshold categorises these cases as -1 (not bleed). This classifying of 50/50 cases as bleeds is an attempt to err on the side of caution, which is appropriate given the intended clinical application. Hence, this subtle change causes a reduction in specificity in these cases.

### 3.5 Study 4: Effect of electrode positioning

In this study, the positioning of the electrode ring is varied, while all other variables are kept constant. In all training and test cases, 60 dB simulated noise is added to the frames. The classifiers for both unaltered and sorted frames are trained on a data set of 640 measurement frames. Each data set comprises 320 measurement frames from the base numerical model and 40 measurement frames from each the 8 combinations of the 30 ml and 60 ml bleeds at each of the 4 possible locations added. Both classifiers, when trained returned an AUC value of 1.

**Fig 8:**
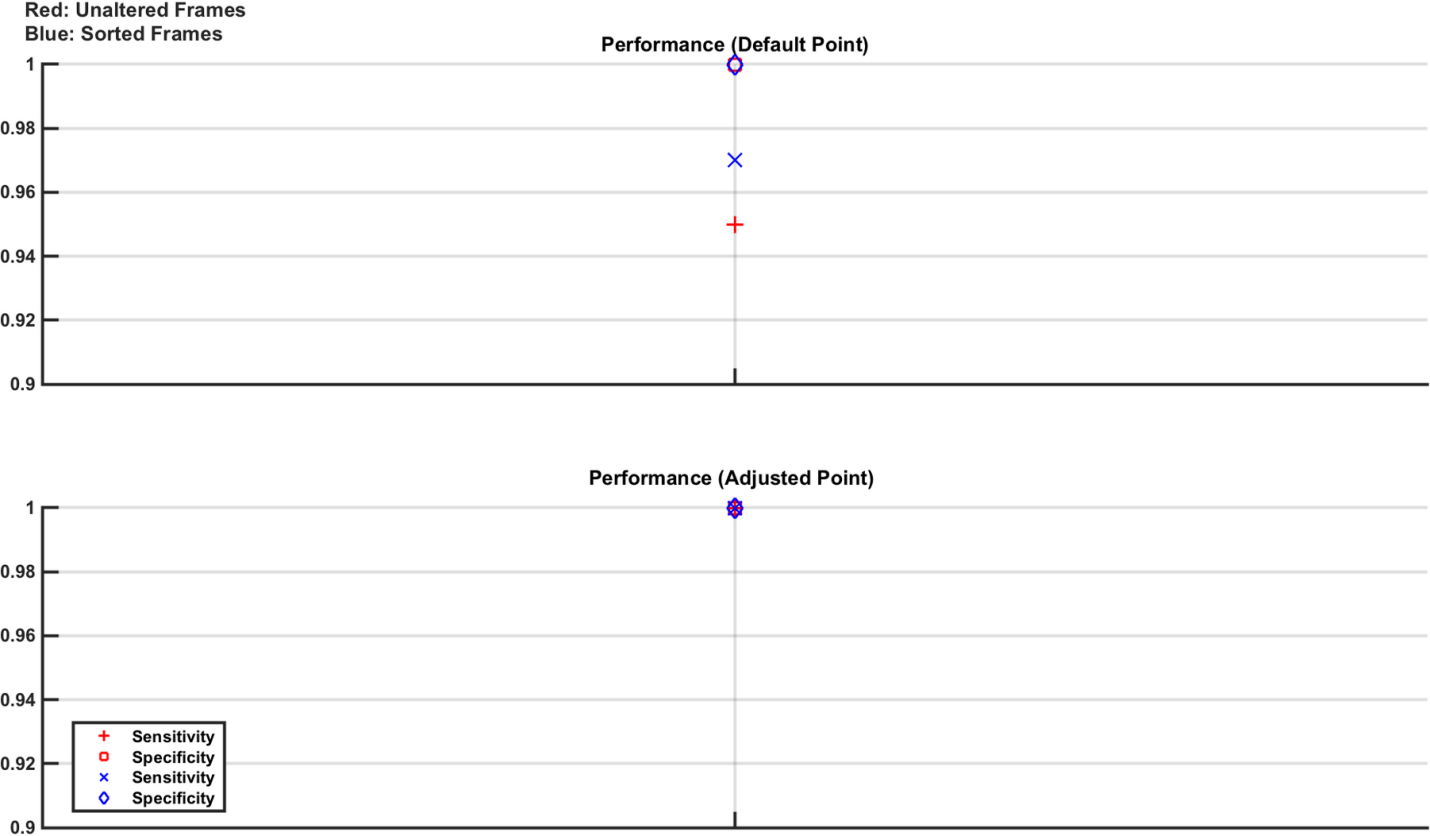
Classifier results from test data in numerical model Study 4: Effect of electrode location. The models are trained using frames from the base numerical model as the normal and eight lesion models produced from the normal and all combinations of the 30 ml or 60 ml lesion at one of the 4 locations. The frames are contaminated with noise at 60 dB SNR. As usual, two classifiers are produced, one trained with unaltered frames and one with sorted frames. These are then tested with the electrode ring placed at one of two possible locations differing from, and parallel to, the original by ± 2 mm. The results show that classifier performance, though excellent, is noticeably affected by even these small changes in electrode positioning. Using the adjusted operating point however, mitigates against the reduction in sensitivity introduced by the electrode positioning change.

Next these models, one normal and 8 lesion, have the electrode ring positioned at one of the locations that deviate from the original training position by ± 2 mm, such that the plane of the ring is parallel to the original. These position changes model user error in positioning of the ring. Each new normal model is used to produce 40 measurement frames and each new lesion model used to produce 5 measurement frames, giving a testing set of 80 normal and 80 abnormal measurement frames. The results from the performance of these single test sets are shown in Fig 8.

The intention of this study was to mimic a minor error in electrode positioning. Even such a minor movement causes a noticeable drop in performance. The results fall from 100% sensitivity and specificity to a value of 95%+ in all cases using the default operating point; however, this drop demonstrates that even mm changes can cause challenges in EIT. However, using the adjusted operating point, designed to increase sensitivity, moves sensitivity back to 100% again without affecting specificity, thus mitigating the effects of the 2 mm positional error. This result demonstrates the value of the adjusted operating point in scenarios such as this.

### 3.6 Study 5: Effect of anatomy

This study examines the ability of the classifier to detect lesions in head and brain anatomies different to the one the training data set is recorded from. All frames used in training and testing had 60 dB noise added. The training models are comprised of the base numerical model as the normal, with 8 lesion models produced from the normal using all 8 combinations of the 30 ml and 60 ml lesion at all locations. In total 5,120 normal and 5,120 lesion (640 from each lesion model) measurement frames were used to train the classifier. The resultant classifiers trained from the unaltered or sorted measurement frames both reported an AUC value of 1.

**Fig 9:**
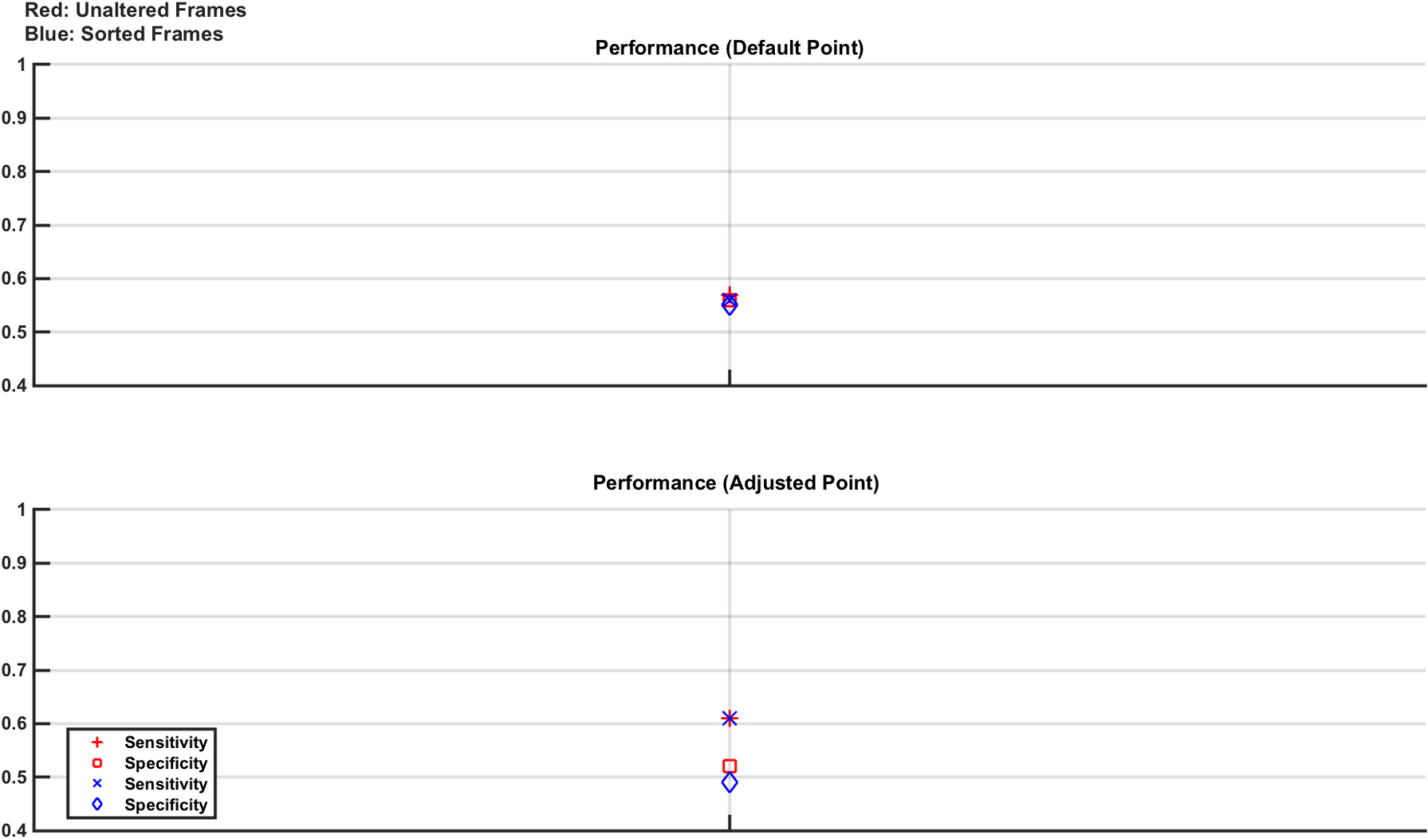
Classifier results from test data in numerical model Study 5: Effect of anatomy. Performance is generally poor in all cases with sensitivity and specificity close to 0.5, which is the value a random classifier would perform at. Analysis showed that an excess of brain tissue or lack of outer tissue in a test model compared to the ratio in the base numerical model used in the training set often confounded the classifier resulting in a “bleed”. Similarly, a lack of brain tissue or excess of outer tissue with respect to the base numerical model resulted in a classification of “normal”. In some cases, the 60 ml lesion gave a correct classification result, but in general a variance in tissue ratio away from that used in the training models usually confounded the classifier.

Next the 80 different normal models, produced as explained in section 3.1, using the 9 different head and 9 different brains, with the same electrode ring height, were each used to create 16 test normal frames each. Each of these normal models were then combined with each of the 8 possible lesions to create 640 lesion cases with 2 frames from each used to complete the test set. Hence, the classifier is trained on lesions in one anatomy but tested on lesions in a variety of 80 different anatomies. Fig 9 shows the performance of the two respective classifiers after inputting the test data.

The results show that anatomy has a profound effect on classifier performance with sensitivity and specificity in all cases near to a value of 50% which is what a random classifier would perform at. Deeper analysis of the results revealed that in cases where the ratio of outer tissue to brain tissue of a test model was larger than that of the base numerical mode (around which the training sets were developed) the classifier usually returned a value of “normal” whereas when this ratio was lower than the base numerical model a value of “bleed” was returned. In some cases, the larger lesion size could be correctly classified despite anatomical differences, but in general the classifiers were excessively sensitive to differences in anatomy of the test models compared to that used to train.

### 3.7 Study 6: Final overall study

As a final overall analysis, a series of tests are done featuring all 243 normal models and 1944 lesion models. The base STL files of the head and brain, as described in section 3.1, are each distorted by ± 5% in each of the X, Y, Z axes separately as well as in all 3 axes simultaneously, resulting in 9 distinct head and brain models and hence 81 different combinations. The 16-member electrode ring was then placed at 1 of 3 distinct locations giving 243 models of the normal. To create the lesion models, each of these 243 normal models were combined with one the 30 ml or 60 ml haemorrhages at one of the 4 locations. All permutations of these resulted in the 1944 lesion models.

**Fig 10:**
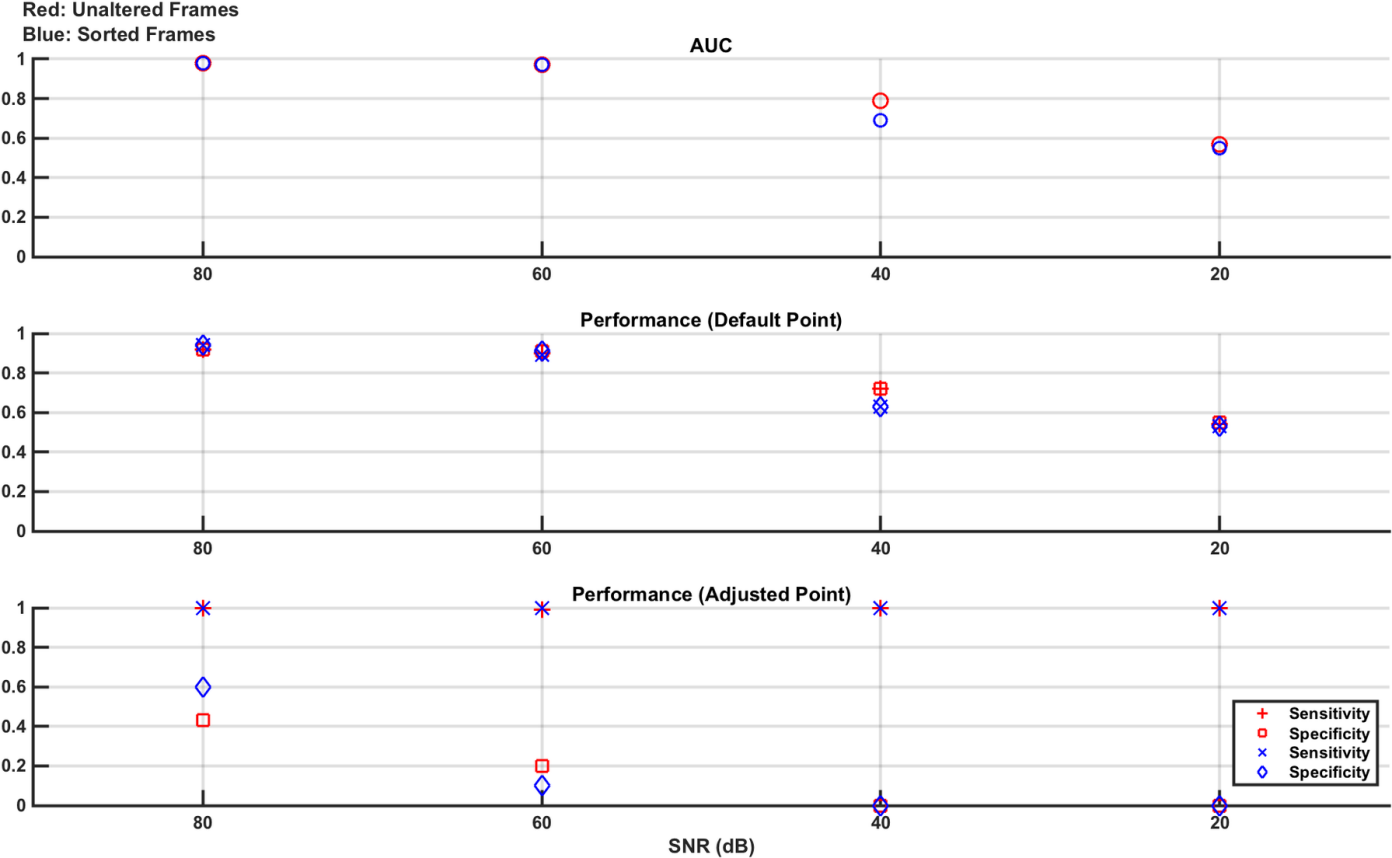
Classifier results from test data in numerical model Study 6: Final overall study. The classifiers perform well at the 80 dB and 60 dB points. The sorting of the frames, developed to compensate for test data featuring lesions in previously unseen locations, has little effect as training features cases from all 4 locations. However, there is a noticeable improvement when using sorted frames at the 80 dB point. Using the adjusted operating point increases sensitivity in all cases at the expense of a perhaps unacceptable drop in specificity.

Training and test sets comprised 139,968 and 15,552 measurement frames, respectively. In each case, the sets are evenly divided between frames from the normal and lesion models, with each of these two subsets further divided to ensure an identical number of frames are provided by each model contributing to each set. Hence, each of the 243 normal models each contribute an equal number of frames totalling 69,984 for the training set and 7,776 for the test set, and likewise for each of the 1944 lesion models. Measurement frames are again used either unaltered or sorted, resulting in two classifiers. This experimental setup is repeated for each of 80 dB, 60 dB, 40 dB and 20 dB noise levels. The AUC of the trained classifiers, and the sensitivity and specificity of the classifiers on the test sets are reported in Fig 10.

The classifiers perform well at the 80 dB and 60 dB noise levels with a subsequent drop in AUC and sensitivity/ specificity results seen at the 40 dB level and the classifier performing little better than random at 20 dB SNR. There is little difference in the results produced from using the unaltered or sorted frames, except at the 80 dB level where the sorted frames result in a small but noticeable improvement. The sorted frames were developed to compensate for the challenges in encountering lesions in the test sets at locations not seen in the training set, but this is not the case in this study. Using the adjusted operating point does boost sensitivity but causes a perhaps unacceptable drop in specificity, resulting in a drop in false negatives but maybe an excessive level of false positives. This overall study indicates the system performs well provided the SNR does not drop below 60 dB, and ideally at 80 dB or better. Further, the sensitivity/ specificity balance seems to be the best at the default operating point; however, if the motivation is to eliminate false negatives in order to be confident that bleed cases are not misclassified (which is crucial in stroke diagnosis), then the adjusted operating point may be preferable. It is seen that the performance at the adjusted operating point for the sorted frames at 80 dB has a sensitivity of 1 and a specificity of ~0.6. Presumably moving to 90 dB or higher would increase this specificity, making an even stronger argument for working at the adjusted operating point.

## 4. Classification experiments on a phantom model

This section describes studies carried out on a phantom model. These studies were analogous, as much as possible, to those performed with the numerical models in section 3. One phantom was fabricated, and an EIT system, the Swisstom EIT-Pioneer set [37], was used to produce all measurement frames, operating at the noise level inherent to the setup. Hence, it was not possible to fully replicate the variety of models and noise levels used in numerical experiments. However, even with this limitation, a series of 5 experiments were performed including an initial study examining repeatability before analysing the effect of location, size, electrode placement variation and a final overall experiment. The phantom and hardware are briefly described before each study is outlined with results reported and discussed.

### 4.1 The phantom model & EIT hardware

Tissue mimicking materials, composed of variable amounts of graphite, carbon black, and acetone in a polyurethane base are used to produce mixtures that emulate target conductivity values of 0.1 S/m and 0.7 S/m. The former relates to the value assigned to the outer layer of the head, modelled as a weighted aggregate of all tissues external to the brain, while the latter relates to the target value of blood. The derivation of these values for these tissues, at a typical EIT frequency point of 50 kHz [38], was previously described in [35]. Further, the head phantom is fabricated in the manner described in [35], with the exception of the inner brain layer being left hollow in this study. This cavity is filled with a 0.03 M saline solution which has a conductivity of approximately 0.3 S/m hence emulating aggregate brain tissue [39]. It is noted that the STL files used to produce 3D printed moulds for the head and brain are the same as those used to produce the base numerical model. The mixture emulating blood is used to fabricate solid spheres of 30 ml and 60 ml volume which are suspended into the saline brain layer to model brain haemorrhages.

The EIT hardware system used is the Swisstom EIT-Pioneer set, a commercially available system for research [37]. A ring of 16 EEG electrodes are placed on the head phantom using electrolyte gel [40], symmetrically across the sagittal plane, as in the numerical study. The electrodes are connected to the Pioneer set, which is connected to a laptop. Before use, the Swisstom set is allowed to warm up for about an hour. Measurements are then recorded, using the same “skip 2” pattern used in the numerical experiments, at 10 frames per second and at 50 kHz, for the different phantom setups described in each experiment. The Swisstom EIT-Pioneer requires 32 electrodes be connected, but by connecting the electrodes to the set in a particular way and then parsing the frame correctly, the frame for the 16-member ring is extrapolated out. Data was collected from the phantom on 3 consecutive days, with the electrodes being repositioned on the phantom head on each day. The saline-filled head, without lesions, constituted the normal. Over the course of the daily measurements approximately 24 cases of the normal were taken, of about 70 s each in duration giving ~17000 normal frames daily. These frames were used to calculate an approximate figure for the signal to noise ratio (SNR) of the system, calculated as the ratio of the mean to the standard deviation of the values for each measurement channel for all the frames. The average SNR over the 3 days was approximately 50 dB.

**Fig 11:**
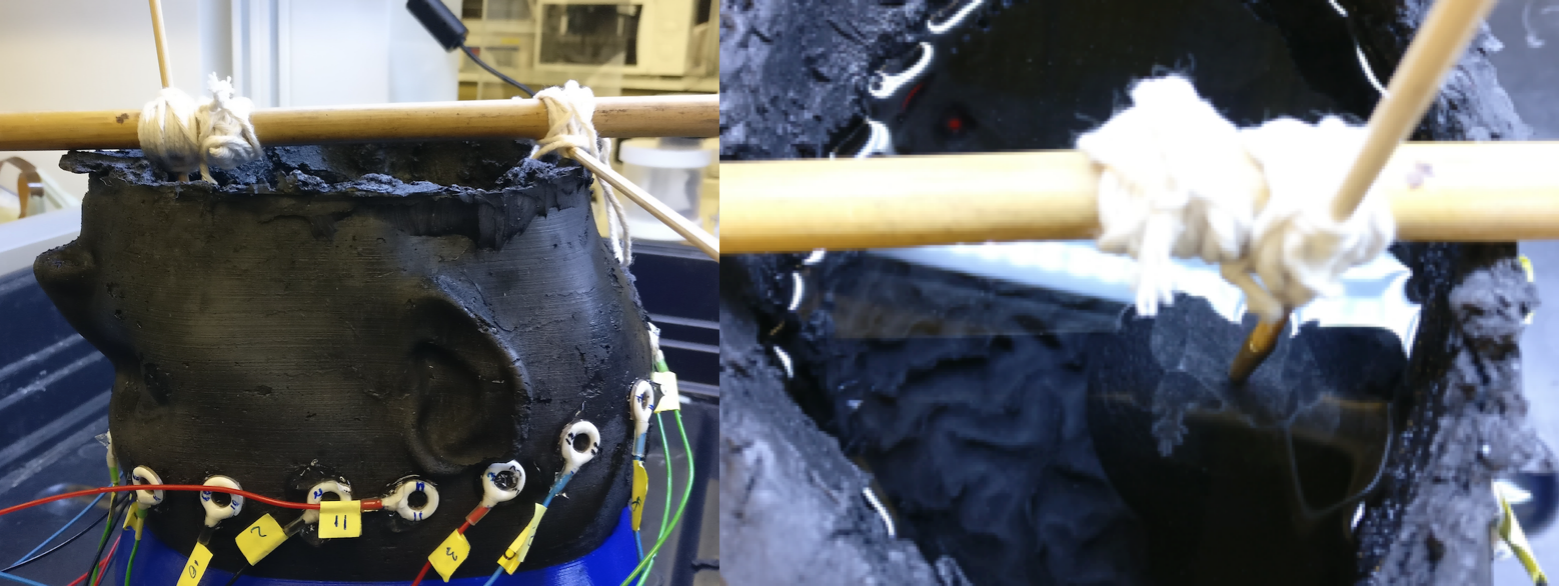
Left: The phantom head model with EEG electrodes attached to form a 16-member ring. Right: Wooden rods are used to attach and place lesions inside the saline brain layer. The electrodes are attached to a Swisstom EIT-Pioneer set which records measurement frames.

In Fig 11, the phantom setup with EEG electrodes attached is shown. This figure also shows a phantom lesion being suspended into the saline brain layer, with the aid of wooden rods.

### 4.2 Study 1: Repeatability

**Fig 12:**
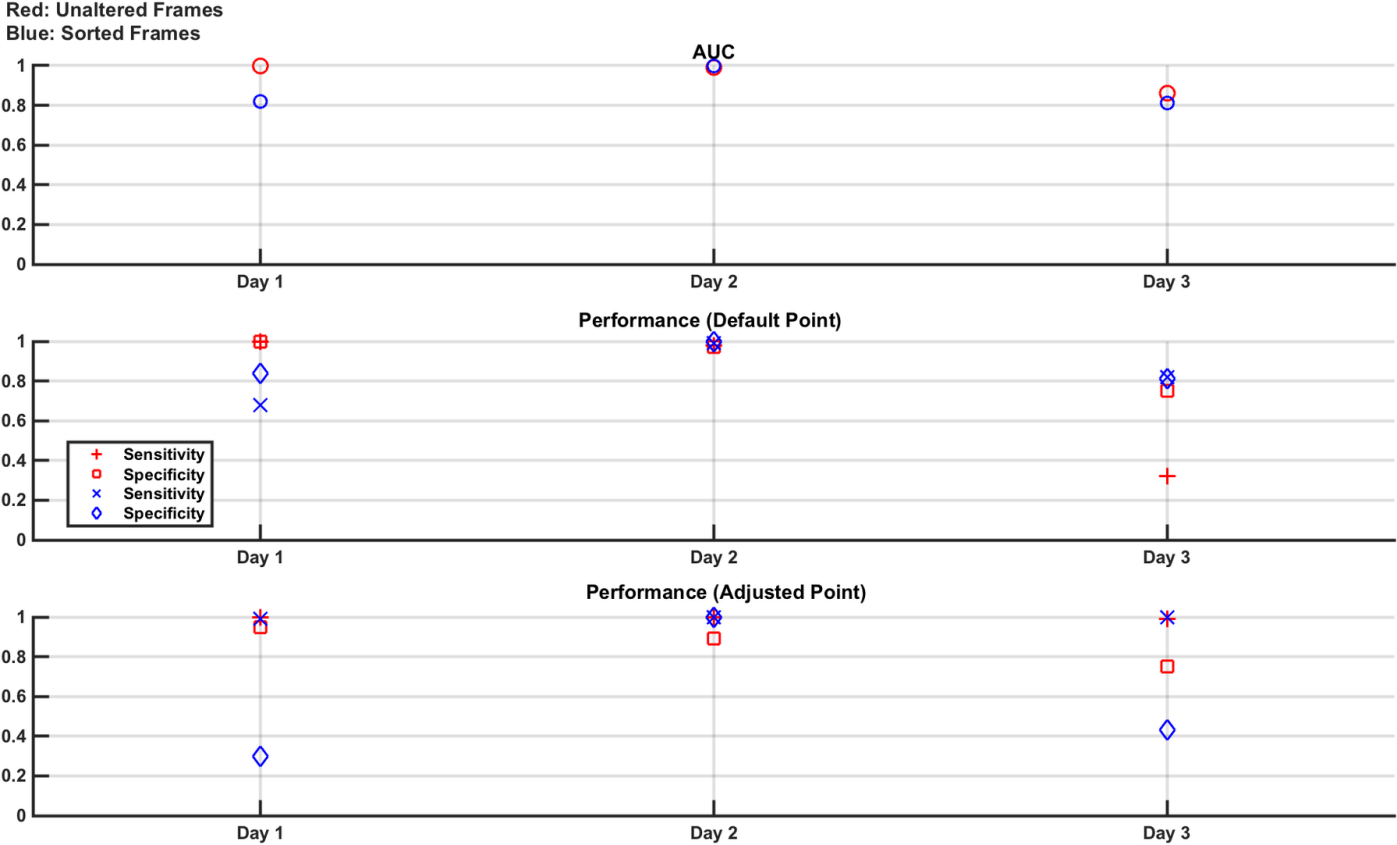
Classifier results from test data in phantom model Study 1: Repeatability. **Variances** are seen in performance of the same experiment on each of the 3 days. Sensitivity approaches 1 in all cases when the adjusted operating point is used, with a variable reduction in specificity depending on the day.

In this study, the ability to achieve repeatable results on different days is assessed. Measurement frames are taken using the setup described above in section 4.1, with the brain layer free of lesions (normal) or with either the 30 ml or 60 ml lesion in the north location. 6 recording sets are taken of the normal case and 3 sets are taken of each of the two lesion cases. The order of recording the sets is randomised and each set is approximately 70 seconds (700 frames) in length. The experiment is performed on 3 separate days, with the electrode ring placed each day and the phantom re-filled with saline. For the frames produced from each day, linear SVM classifiers are trained with 6,800 randomly selected frames either unaltered or sorted (3,400 normal and 1,700 from each of the two lesion cases) and then tested with 800 unseen frames again evenly selected as 400 normal and 200 from each lesion case and either unaltered or sorted. The AUC of the trained classifiers is reported, along with the performance on the test data. These results are shown for each of the 3 days experiments in Fig 12.

The results show a variance in the performance for each of the three days, however the AUC is always > 0.8 and sensitivity approaches 1 in all cases if the adjusted operating point is used. However, using the adjusted operating point has a negative impact on the specificity, the magnitude of which varies between days. The differences in results over the 3 days show the challenge in achieving repeatability using an EIT system, which is sensitive to effects such as changes in electrode contact and positioning which may vary day to day and over the course of a day as the contact gel dries out [41].

### 4.3 Study 2: Effect of lesion location

**Fig 13:**
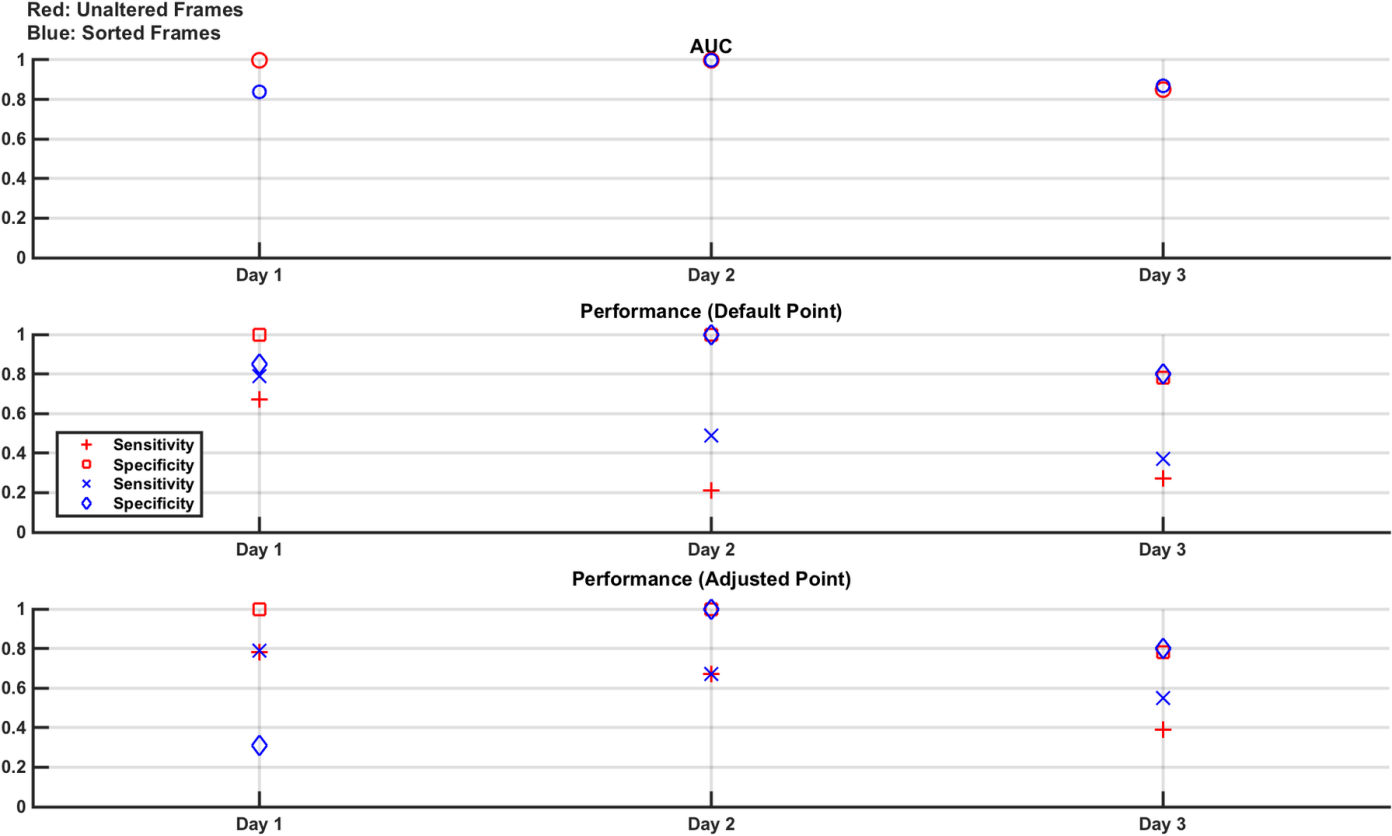
Classifier results from test data in phantom model Study 2: Effect of lesion location. On average, the best overall performance is seen when the frames are sorted, and classification performed at the adjusted point designed to maximise sensitivity. Results are seen to differ from day to day as expected due to variation in electrode positioning and other sources of systematic error.

This study is analogous to that described in Section 3.3, where the performance of the classifier when tested with lesions placed in locations different to that used in the training set is assessed. Training sets are comprised of 7,800 measurement frames made up of equal amounts of normal and lesion models. The lesion models are the 30 ml or 60 ml bleed in the north location, with an equal amount of each making up the lesion measurement frames. The test set is comprised of 1,200 measurement frames of which 600 are unseen normal measurement frames and the remaining lesion measurement frames composed of 100 each from all combinations of the 30 ml and 60 ml bleeds at each of the other 3 locations of east, south and west. The measurement frames sets are taken as 70 s recordings of each lesion case, repeated 3 times; the normal case is repeated 24 times. All measurements are taken in random order, with random measurements selected for training and testing (with no overlap). The complete experiment is repeated completely from the start on 3 consecutive days. The training and test measurement sets are again used unaltered or sorted with the results shown in Fig 13.

The results again show the variance in day to day repeatability of the experiments on a real-life phantom. On average over the 3 days, the best overall performance is seen when the frames are sorted, and classification is performed at the adjusted operating point to maximise sensitivity. This conclusion is consistent with the results observed in Fig 5. The sorting of the frames should, theoretically, offer a robustness to differences in lesion location and while this effect is not as pronounced as in the numerical model, it is observed in this equivalent phantom experiment.

### 4.4 Study 3: Effect of lesion size

**Fig 14:**
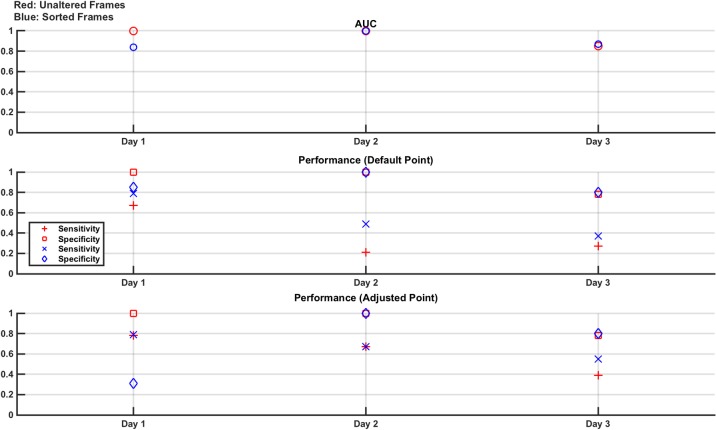
Classifier results from test data in phantom model Study 3: Effect of lesion size. Specificity is high in all cases, which is a consequence of the normal model being the same for the training and test measurement frames. Sensitivity is boosted using the adjusted point reaching about 80% using the results from Day 1 but dropping to under 60% for Day 3.

This study, similar to that described in the first part of Section 3.4, examines the classifier performance in detecting lesions smaller than those trained with. The classifiers are trained with normal measurement frames and measurement frames recorded from the phantom with the 60 ml lesion at one of the 4 positions. The trained classifiers are then tested on unseen normal measurement frames and measurement frames from when the 30 ml lesion is placed at one of the 4 positions. The training set comprises 14,800 measurement frames and the test set comprised 1,600 measurement frames, with each evenly divided between normal and lesion measurement frames with the lesion measurement frames evenly split between all 4 positions. The measurement frames are gathered in the same way as described in Section 4.3. The measurement frames are used either unaltered or sorted. The results are shown in Fig 14.

As a result of the normal being the same for both the training and test measurements (aside from frame to frame changes in random measurement noise), specificity should be, and is, high. Sensitivity is low if using the default operating point but rises to about 80% when using the adjusted operating point for the data taken on Day 1, dropping to 60% on the Day 3 data. These results imply a challenge in detecting smaller volume lesions in the phantom than those trained on.

### 4.5 Study 4: Effect of electrode positioning and anatomy

**Fig 15:**
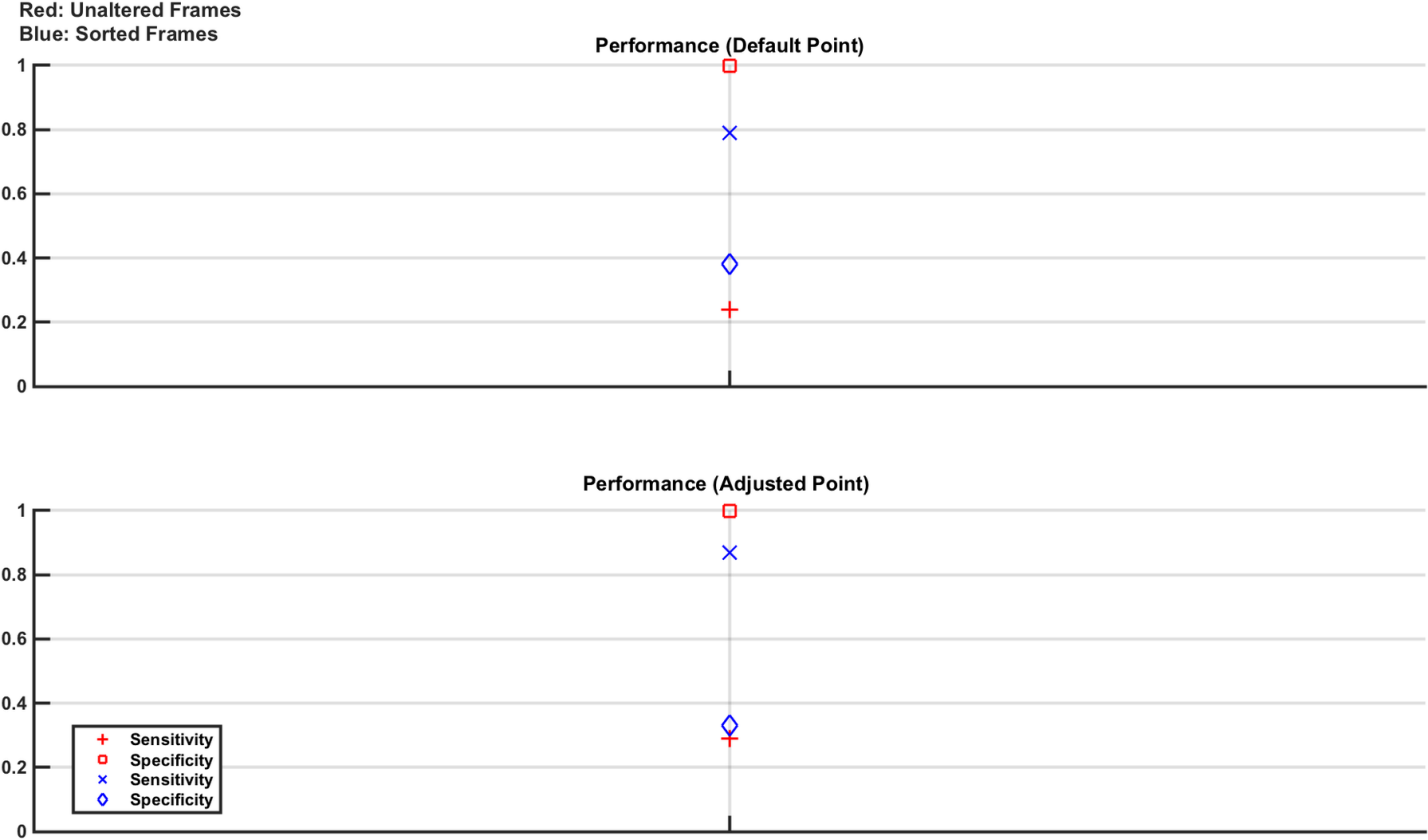
Classifier results from test data in phantom model Study 4: Effect of electrode positioning and anatomy. Any given classifier performs poorly with simultaneous high values of specificity and sensitivity not achieved in any case. Interestingly, the classifier trained and tested with unaltered frames give a specificity of 100%, while the classifier trained and tested with sorted frames give a sensitivity of near 100% suggesting that a cascade of classifiers may provide improved results.

In this study, measurement frames from normal and lesion cases taken on one day are used to train with measurement frames from a different day used to test. Since the platform was setup anew each day, this experiment models both changes in electrode positioning (due to the physical inability to position the electrodes in precisely the same location from day to day) and also different anatomies, since the electrodes will be in contact with a different part of the phantom on the different days. On a given day, the 8 lesion cases (all combinations of the 30 ml and 60 ml lesions in all 4 locations) have approximately 70 s recordings taken 3 times, with 24 normal recordings of the empty saline brain also taken. The order of the recording is randomised. Again, the measurement frames are used either unadjusted or sorted. In total, 14,400 measurement frames are used to train with 1,440 used to test. The sets are evenly divided between normal and lesion cases (and evenly divided between the different lesion cases). The trained classifiers reported a AUC value of 1, with the performance results on the test data shown in Fig 15.

The results show that the classifiers failed to give simultaneously high specificity and sensitivity. However, interestingly the classifier trained with unaltered frames gave a specificity of 100% while the classifier trained with sorted frames gave a sensitivity of near 100%. This result opens the future possibility of implementing a cascade classifier approach to give more robust classification in scenarios such as this.

### 4.6 Study 5: Final overall study

**Fig 16:**
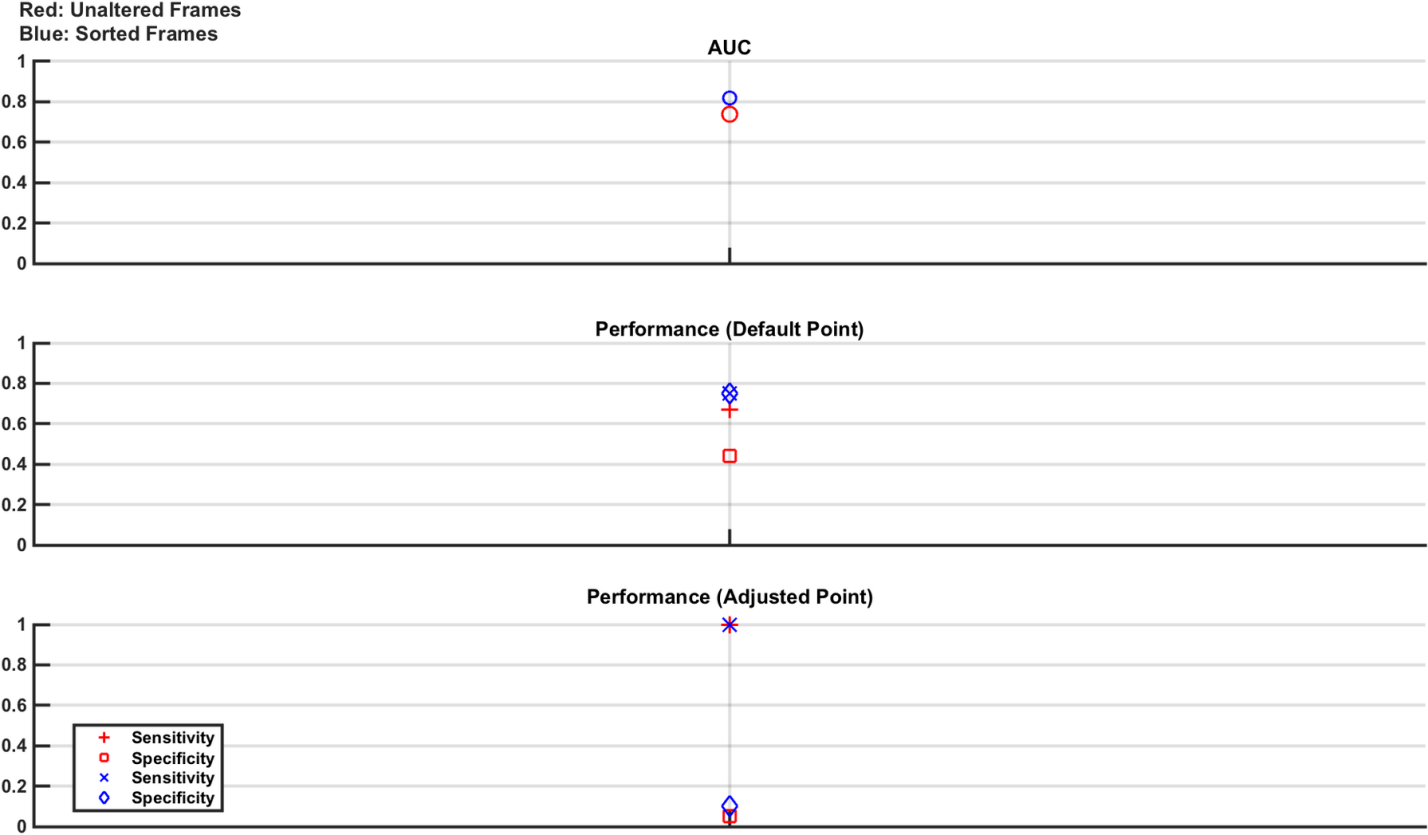
Classifier results from test data in phantom model Study 5: Final overall study. The best performance is seen with the sorted frames used at the default operating point, giving a sensitivity and specificity of 75%. Moving to the adjusted operating point improves sensitivity but severely causes a reduction to specificity. The results are between those of the 60 dB and 40 dB numerical study results reported in Fig 10, as expected since the experimental SNR is ~50 dB.

The final study trains and tests from the combined pool of measurement frames from all measurements conducted over all 3 days. The training set is comprised of 28,800 measurement frames, with the test set comprised of 2,880 measurement frames. The frames in each set are evenly selected from each of the 3 days, evenly divided between normal and lesion cases and with the lesion frames evenly split between all 8 lesion scenarios. No frame in the training set is repeated in the test set. As usual, two classifiers are trained and tested–one using unaltered frames and one using sorted frames. The results, shown in Fig 16, show that the best achievable performance is about 75% sensitivity and specificity for the case of sorted frames used at the default operating point. Using the adjusted operating point improves sensitivity but causes a severe drop in specificity. Interestingly, the results in Fig 16 lay between those of the 60 dB and 40 dB numerical results as seen in Fig 10. The SNR of the phantom experimental platform was calculated at about 50 dB, which is in line with these results.

## 5 Performance of other classifiers

In previous sections, the focus was the examination, as proof of concept, of the use of EIT measurement frames with a linear SVM classifier and the effect of changing variables related to the clinical application (such as lesion parameters and anatomical parameters) on classifier performance. A linear SVM offers advantages such as speed, low computational cost and ease of implementation. However, given the nature of EIT and the measurement frames produced, a linear kernel may not be best choice of classifier for the intended application. This classifier choice was not the focus of this study but is an important consideration for future development of the technology and is thus discussed briefly here.

In related studies, a popular kernel for SVMs is that of a Radial Basis Function (RBF) [18,19] which is a non-linear function that defines the separating hyperplane. Another popular machine learning algorithm is that of neural networks (NN), which unlike SVMs are unsupervised classifiers based loosely on a model of the brain and feature ‘neurons’ as processing and learning layers between the input and output [42]. These two alternative classifiers were compared to that of the linear SVM using the data of the two final overall studies from both the numerical (Section 3.7) and phantom (Section 4.6) studies. These final overall studies featured pooled data with maximisation of parameter variability in both normal and lesion cases. Using the numerical set the classifier comparison study was performed at 80 dB, 60 dB, 40 dB and 20 dB SNR while the phantom set facilitated one classifier comparison study at the SNR point of the Swisstom EIT-Pioneer which was found to be about 50 dB. In these classifier comparison studies, the EIT measurement frames were used as sorted frames and the default operating point was used.

In previous subsections, the performance metrics were reported for the trained classifier on one set of unseen test data. In this section, however, the performance metrics of sensitivity, specificity and accuracy are given for each of the three classifiers as a mean and standard deviation of ten separate test data sets.

For the linear and RBF kernel SVM classifier results, a ten-fold testing scheme was used to obtain the averaged performance metrics. The data set is first split with 90% used for training and 10% held out as a test data set. The training data set is used to optimize the classifier; ten-fold cross-validation is performed with a Bayesian optimization protocol to obtain the optimized box constraint and kernel scaling factor. The optimized parameters are those that lead to the lowest generalized error in the ten-fold cross validation. These optimized parameters are used to train a final classifier that is then applied to the test data set. This process is repeated ten times until all data has been used as part of the test data set. The averaged results (across these ten runs) of the performance of the final classifier is presented to give a more generalizable expectation of the classifier performance.

In the case of the NN classifier, the data is divided 70%:15%:15% into training, validation, and test data. Ten hidden neurons are used and a scaled conjugate backpropagation algorithm applied. The confusion matrix generated from the test data allows calculation of the sensitivity, specificity and accuracy. This process is repeated ten times.

The sensitivity, specificity and accuracy statistics from the three classifier types are compared below in Table 1. Altogether, there are 5 types of data compared in Table 1: 4 based on the total numerical data with different SNRs, and 1 based on the total phantom data.

**Table 1:** Performance results across classifiers. The sensitivity, specificity and accuracy of each of three classifiers (Linear SVM, RBF SVM, NN) is expressed as the mean ± standard deviation following training and testing of each classifier on the total pooled numerical data (at the four SNR levels of 80 dB, 60 dB, 40 dB and 20 dB) and the total pooled phantom data.

	Numerical Data				*Phantom Data*
Classifier/ Noise	80 dB	60 dB	40 dB	20 dB	*~ 50 dB*
**Linear SVM**					* *
Sensitivity	0.80 ± 0.42	0.69 ± 0.42	0.52 ± 0.39	0.45 ± 0.44	*0*.*90 ± 0*.*32*
Specificity	0.60 ± 0.52	0.79 ± 0.42	0.60 ± 0.37	0.55 ± 0.44	*0*.*70 ± 0*.*48*
Accuracy	0.69 ± 0.27	0.73 ± 0.24	0.55 ± 0.07	0.49 ± 0.02	*0*.*79 ± 0*.*27*
**RBF SVM**					* *
Sensitivity	1.00 ± 0.00	0.99 ± 0.01	0.65 ± 0.05	0.52 ± 0.24	*1*.*00 ± 0*.*00*
Specificity	1.00 ± 0.00	0.97 ± 0.01	0.62 ± 0.04	0.51 ± 0.24	*0*.*99 ± 0*.*00*
Accuracy	1.00 ± 0.00	0.98 ± 0.01	0.63 ± 0.03	0.51 ± 0.03	*0*.*99 ± 0*.*00*
**NN**					* *
Sensitivity	0.99 ± 0.00	0.97 ± 0.02	0.66 ± 0.03	0.50 ± 0.05	*1*.*00 ± 0*.*00*
Specificity	0.99 ± 0.00	0.96 ± 0.02	0.59 ± 0.04	0.53 ± 0.05	*1*.*00 ± 0*.*00*
Accuracy	0.99 ± 0.00	0.96 ± 0.01	0.63 ± 0.03	0.51 ± 0.02	*1*.*00 ± 0*.*00*

It is evident from Table 1 that both the RBF SVM and NN offer significantly better sensitivity, specificity and accuracy than does linear SVM at the 80 dB and 60 dB SNR values when using the numerical data set and also on the phantom data set. Additionally, the RBF SVM and the NN classifiers offer superior robustness to a linear SVM as noted by the much smaller standard deviation in all metrics across all test cases. Performance for all classifiers starts to significantly degrade once the SNR falls to 40 dB or lower. This gives insight on the minimum dynamic range expected with a physical system to allow for successful implementation of a classification algorithm. Interestingly, the two alternative classifiers offer excellent performance with the phantom data, which was produced with an SNR calculated at about 50 dB. These results highlight that both an optimized RBF kernel SVM and a NN classifier can be used to classify EIT measurement frames for brain bleed detection.

## 6 Discussion and conclusions

This paper has sought to explore the feasibility of using measurement frames designed for EIT, traditionally used to reconstruct images, as data sets for a SVM classifier. A key motivation for this study is the need to tackle the challenges faced by EIT systems in imaging static or quasi-static scenes. An example of such a scene would be the case of a brain haemorrhage in a stroke or traumatic brain injury patient, and the realisation that in these cases an image may not be required. In such scenarios, the use of trained classifiers may be sufficient to adequately identify the presence or absence of a haemorrhage, allowing the correct treatment path to be initiated. This work, uniquely, looks at complete EIT measurement frames as the input for a classifier, and for the first time, uses this technique applied to static scenes. A linear SVM was used for most of the study as a fast, easy to implement and computationally cheap classifier. However, two other classifiers (a RBF SVM and NN) were also examined.

A thorough set of experiments have been described exploring the effect of a variety of variables in both numerical and phantom models of the head and brain. In numerical models the effect of noise, lesion location, lesion size, electrode positioning, and anatomy were individually studied and then collectively assessed. Then in the physical phantom model, repeatability, lesion location, lesion size, electrode positioning, and anatomy were individually and then collectively studied.

Valuable insights as to the viability of, and future challenges facing, the technique were found. The SNR of the phantom system, calculated at 50 dB, was below the 60 dB mark which was the approximate threshold for better results in the numerical studies when using a linear SVM. However, it may be the case that such a system may suffice with the selection of a superior classifier as indicated by the performance of the RBF SVM and NN on the phantom data in contrast to the linear SVM. However superior systems with an SNR in the region of 80 dB or higher exist and may, coupled with an optimum classifier, give further improvement in performance especially in challenging scenarios [43]. Further, the numerical studies demonstrated that: sorting the frames by value resulted in a robustness to detecting lesions in previously unseen locations, lesions of size not in the training set were better detected if they were larger than those in the training set, and small changes in electrode placement did not affect results. However, the classifiers were very sensitive to measurement frames from new anatomies, leading to a need to train on a large number of different anatomies. Phantom experiments added more variables associated with a more realistic platform highlighting challenges such as repeatability from day to day, but did give results that fit with those of the numerical experiments. In this work, a goal was maximal sensitivity to brain haemorrhage detection since occurrence of false negatives, especially in the case of candidate alteplase stroke patients, may be fatal. The use of an adjusted operating point, designed to maximise sensitivity, was effective but with a cost to specificity.

This initial study has demonstrated the feasibility of using EIT measurement frames to identify bleeds in the brain. For all investigated classifiers on phantom data, a specificity of greater than 90% was achieved. A comparison of the sensitivity, specificity and accuracy of linear SVM, RBF SVM, and NN on the pooled total numerical and phantom data shows the latter two classifiers offer significantly better performance, with the RBF SVM and NN notably achieving 100% sensitivity on phantom data.

A future study may look at the use of such classifiers in a cascade, at the original operating point and the adjusted operating point, with the aggregate result perhaps being of value. The measurement frames in this study were either unaltered or sorted but other pre-processing possibilities exist such as filtering to reduce noise, weighting of data from select channels, and so on. Future work will also look at better numerical and phantom platforms in terms of realism, complexity of test scenarios and hardware. A study dedicated to classifier choice and optimisation should also be undertaken, and may lead to further performance improvements. A challenge is and will be matching the performance of numerical models in real world phantoms and patients.

EIT has the potential to be a valuable diagnostic aid, even in cases without changes occurring within the time frame of interest. The application of classifiers to EIT measurements is an emerging and novel approach which shows promise and is worthy of further investigation.
